# Flagellar glycosylation with pseudaminic acids is widespread in the genus *Clostridium*

**DOI:** 10.1186/s12866-026-04975-z

**Published:** 2026-05-06

**Authors:** Orlagh H. Anderson, Jay M. Johnson, Emily K. P. Flack, Martin A. Fascione, James P. J. Chong, Gavin H. Thomas

**Affiliations:** 1 Department of Biology, University of York, Wentworth Way, York, YO10 5DD UK; 2 Department of Chemistry, Green Chemistry Centre of Excellence, University of York, Heslington, York YO10 5DD UK

**Keywords:** *Clostridium butyricum*, Flagellar glycosylation, Hypervariable region, Legionaminic acid, Pseudaminic acid

## Abstract

**Background:**

The ability of bacteria to chemically modify their cell surfaces through *O*-linked glycosylation is well characterised in Gram-negative bacteria and includes the modification of flagellin proteins with nonulosonic acids (NulOs). These modifications are widespread and have established roles in flagellar assembly, motility and virulence in various pathogens. However, there are limited documented examples of similar modifications in Gram-positive bacteria, and the significance of these is less well understood. In this study, we expand upon our previous findings that the flagellar biosynthetic locus of several pathogenic *Clostridium butyricum* strains contains a cluster of genes predicted to be involved in the biosynthesis of NulOs, and examine similar clusters across the whole genus *Clostridium* to assess the wider occurrence of NulO-mediated flagellin modification.

**Results:**

Using orthologues of the components of the biosynthetic pathways for the NulOs pseudaminic acid (Pse) and legionaminic acid (Leg) in the pathogenic species *Campylobacter jejuni*, we predict that the flagellar biosynthetic loci of several pathogenic *C. butyricum* strains also encode genes for these modifications. To verify this, we demonstrate that the predicted PseB orthologue encoded in the flagellar biosynthesis locus of the pathogenic strain *C. butyricum* 5521 is able to catalyse the first step of Pse biosynthesis. Furthermore, we show that across the genus *Clostridium*, genes involved in flagellar glycosylation are located in a hypervariable region (HVR) of the flagellar biosynthetic locus, which is flanked by the flagellar structural genes *flgB* and *fliD*. The content of this region differs considerably across strains and species, but remarkably over half of the *Clostridium* genomes encode NulO biosynthesis genes in these regions. Finally, we present evidence that suggests that this flagellar HVR has evolved independently from the rest of the genome.

**Conclusions:**

We show that genes required for NulO-mediated flagellar glycosylation are widespread across the genus *Clostridium.* Our findings have potential applications in the characterisation of pathogenic strains within these species and in the engineering of strains used in industrial biotechnology.

**Supplementary Information:**

The online version contains supplementary material available at 10.1186/s12866-026-04975-z.

## Background

The bacterial flagellum plays a key role in motility and the adaptation to diverse environments. In particular, it has been recognised that flagella-mediated motility is required by both commensal and pathogenic species for colonisation of the gastrointestinal tract. Amongst Gram-negative examples of these gut-dwelling species, there have been widespread reports of the *O*-glycosylation of flagellin monomers with NulOs and their derivatives. This has a role in a range of processes which include: flagellar assembly, motility, colonisation, host immune invasion, host specificity and biofilm formation [1–6]. For the two major gastrointestinal pathogens *C. jejuni* and *Helicobacter pylori*, it has been determined that flagellar glycosylation with NulOs is essential for flagellar filament assembly and virulence [7–12]. As such, the flagellar glycosylation systems of *C. jejuni* and *H. pylori* have been the most well characterised among Gram-negative bacteria.

*C. jejuni,* the leading cause of foodborne bacterial gastroenteritis, produces amphitrichous flagella composed of the highly similar flagellin monomers, FlaA and FlaB [13, 14]. These are *O*-glycosylated at up to 19 sites on their surface exposed domains, with up to seven different Pse and Leg-based moieties [9, 14–17]. The genes involved in flagellar glycosylation are located in a genomic HVR adjacent to *flaA* and *flaB*, which varies in size depending on the strain. This ranges from ~ 25 kb in the human-associated strain 81–176, to over ~ 50 kb in the livestock associated strains NCTC 11168 and RM1221 [18, 19]. Moreover, the high heterogeneity of flagellin glycoforms has been attributed to the genetic variation in this region, and it has been suggested that this extensive variability provides a range of antigenic profiles, which may serve as an immune evasion strategy [20, 21].

*H. pylori*, a gastrointestinal pathogen associated with gastritis, peptic ulcers and mucosa-associated lymphoid tissue lymphomas, requires flagella-mediated motility for colonisation of the stomach [12, 22]. The unipolar bundle of two to six sheathed flagella is composed of FlaA and FlaB flagellin monomers, which are *O*-glycosylated at seven and ten sites on FlaA and FlaB, respectively [17, 23, 24]. In contrast to the heterogeneity of flagellin modifications observed across *C. jejuni* strains, the flagellins of *H. pylori* are only modified with Pse [17, 25]. This may be due to the presence of the flagellar sheath, which has potentially reduced the evolutionary need to employ Pse derivation as a means of immune evasion [6].

In addition to these two pathogenic species, *Aeromonas caviae*, a causative agent of human gastroenteritis, possesses a relatively simple Pse flagellar modification system and has thus been used as a model organism for the study of this pathway [26]. It has been determined that the flagellum of *A. caviae* is composed of the flagellin subunits FlaA and FlaB, which are *O*-glycosylated with Pse at six to eight sites in their central, surface exposed regions [26]. The genes required for this process reside within a small genomic island, which is flanked by insertion sequences [27]. In particular, the enzyme responsible for the transfer of CMP-Pse onto flagellin in *A. caviae* has been well characterised in vivo [26, 28]. This putative pseudaminyl-transferase, which is encoded by *maf1*, interacts directly with flagellin in the cytoplasm and is required for flagellar filament assembly, but not flagellin export [26, 28]. As a result, flagellar glycosylation is essential for both motility and flagellum-mediated adhesion to host cells [26, 27, 29].

Although flagellar *O*-linked glycosylation is widespread among Gram-negative bacteria, it has only been described in a small number Gram-positive species, which belong to just seven genera, namely: *Bacillus, Butyrivibrio, Clostridium*, *Clostridioides*, *Geobacillus, Listeria* and *Paenibacillus* [6, 30–34]. These include four *Clostridium* species and the species *Clostridioides difficile* (formerly *Clostridium difficile*). The first report of flagellar glycosylation with a Leg-derivative in a Gram-positive bacterium was for the botulism-causing species *Clostridium botulinum* [35]. During the characterisation of flagellar glycans to determine novel strain identification markers, it was established that the flagella of group I and II *C. botulinum* strains are *O-*glycosylated with a range of di-*N*-acetylhexuronic acids, tri-*N*-acetylhexuronic acids and Leg derivatives, at up to seven sites per FlaA1 monomer [35, 36]. Moreover, it was found that the genes required for flagellar glycosylation are located within the flagellar biosynthesis locus, in a region which has been highlighted as a genomic hotspot for recombination and genetic exchange, and is referred to as a flagellar glycosylation island (FGI) [36, 37]. A HVR of the genome within the flagellar biosynthesis locus has also been identified in the common exotoxin producing pathogen, *Clostridium tetani*. This island of 23–29 genes resides between mobile elements and encodes enzymes which are predicted to be involved in flagellar glycosylation [38]. These include components of the Pse biosynthetic pathway, which vary in organisation across strains. In addition to these two pathogenic *Clostridium* species, flagellar glycosylation has been reported in the non-pathogenic, cheese-spoiling species *Clostridium tyrobutyricum* [39]. Through the characterisation of cell surface structures for the development of detection assays, it was determined that the flagellin of *C. tyrobutyricum* ATCC 25755 is modified with a glycan that contains d-glucose and GlcNAc [40]. It has also been determined that flagellin of the biotechnologically useful species *C. acetobutylicum* is modified by a 12 kDa *O-*linked glycan, which bears a terminal sialic acid-like residue [41].

In this study, we aimed to take a systematic approach to identify and compare the HVRs in the flagellar biosynthesis loci across the genus *Clostridium*. We define a conserved location for a HVR between the flagellar structural genes *flgB* and *fliD* that is present in all *Clostridium* species which have intact flagellar biosynthesis loci. While various glycosylation events are predicted, we find evidence that modification of flagellin with NulOs is widely present across the genus.

## Material and methods

### Collection of *Clostridium* genomes

Nineteen complete *C. butyricum* genome assemblies were retrieved from the National Centre for Biotechnology Information (NCBI) genome database on 19th December 2024. The genomes of strains NBRC 13949, SJ1 and TK520 were excluded from the subsequent analysis due to a duplicate entry, an incomplete assembly and a high percentage of frameshifted proteins, respectively. On 13th January 2025, the reference genome assemblies of 39 additional *Clostridium* species were downloaded from NCBI. Up to four additional complete genome assemblies were downloaded for each species, depending on their availability in the NCBI genome database.

### Prediction of gene functions

The functions of genes present in the flagellar biosynthesis loci were predicted using the tools: BLAST, Interpro, RegPrecise and the CAZy database, and guided by the automatic genome annotations assigned by GenBank and RefSeq [42–46]. OrthoFinder v.2.5.2 was used to identify groups of orthologous genes (orthogroups) across the flagellar HVRs of *Clostridium* species with complete genome assemblies, using the default parameters and the genes *flgB* and *fliD* to indicate the boundaries of this region in each genome [47].

### Phylogenetic analyses

A phylogenetic tree was constructed using the complete genome sequences of the 40 *Clostridium* species reference genomes and the genome sequence of *C. butyricum* CDC_51208, in addition to that of *C. difficile* S-0253 for the outgroup. The genomes were annotated using Prokka (prokka/1.14.5-gompi-2022b), and the output.gff files were aligned using Roary (Roary/3.13.0-foss-2022a), with the -*i* parameter set to 50 [45, 48]. This revealed a total of 232 core genes, determined by their conserved presence in all species. The IQ-TREE program (IQ-TREE/2.3.6-gompi-2023a) was then used to infer a maximum likelihood phylogenetic tree based on the core gene alignment, using 1,000 bootstrap replicates [49]. The resulting phylogenetic tree was visualized and edited using the Interactive Tree of Life tool (version 6.9) [50]. Sequence alignments for sequences annotated by Prokka as PseI were performed with Clustal Omega and visualised using the phylogram tool [51].

### Solvents and materials

Solvents used for high-performance liquid chromatography (HPLC) purposes were HPLC-grade. Deuterium oxide was used at 99.9% D atom. All commercially available reagents were used as received and were supplied by Sigma-Aldrich, Fisher Scientific and Carbosynth.

### Overexpression and purification of PseB

*C. butyricum* 5512 PseB was cloned into pETFPP_1 (Biology Technology Facility, University of York) to produce an N-terminally His-tagged PseB-encoding kanamycin resistant plasmid. *Escherichia coli* Tuner (DE3) was transformed with plasmid via heat shock. To overexpress PseB, 10 mL LBkan (Lysogeny broth, supplemented with 50 μg mL^−1^ kanamycin) was inoculated with colonies and cultured overnight at 37 °C, 180 rpm. 2 L LBkan (2 × 1 L, in baffled 2.5 L Erlenmyer flasks) was inoculated with overnight culture to give an initial OD600 of 0.05. At an OD600 of 0.6, overexpression of PseB was induced with the addition of 1 mM isopropylthiogalactaside and cells were cultured overnight at 37 °C, 180 rpm. Cell pellets were harvested via centrifugation (6000 × *g*, 40 min, 4 °C), resuspended in wash buffer (50 mM sodium phosphate, pH 7.4, 400 mM NaCl, 10 mM ꞵ-mercaptoethanol, 20 mM imidazole), supplemented with protease inhibitor and DNase, and lysed on ice via sonication. Clarified supernatant (40,000 × *g*, 40 min, 4 °C) was purified via Immobilized Metal Ion Affinity Chromatography. The supernatant was applied to a 5 mL HisTrap HP column (Cytiva), that was pre-equilibrated with wash buffer. The column was washed with 10 column volumes of wash buffer and protein was eluted with a gradient of elution buffer (50 mM sodium phosphate pH 7.4, 400 mM NaCl, 10 mM β-mercaptoethanol, 500 mM imidazole) over 10 column volumes. 2 mL fractions were collected and analysed by SDS-PAGE and those containing PseB were pooled and dialysed into size exclusion chromatography (SEC) buffer (50 mM sodium phosphate pH 7.4, 200 mM NaCl, 10 mM β-mercaptoethanol) overnight at 4 °C. Samples were flash frozen and stored at −70 °C.

### PseB activity assay in deuterium oxide

Buffers and stock reagents (UDP-GlcNAc and NADPH) were prepared in D_2_O (Sigma-Aldrich, 99.9 atom % D). 1 mL of SEC purified PseB was dialysed overnight at 4 °C, into 200 mL of deuterated buffer (50 mM sodium phosphate, 200 mM NaCl, 1 mM ꞵ-mercaptoethanol). 800 μL samples that contained 15 μM PseB, 5 mM UDP-GlcNAc and 5 mM NADPH, such that D_2_O:H_2_O was 60:40, were prepared in triplicate. Samples were incubated at 37 °C for 20 h and analysed via high performance liquid chromatography-electrospray ionisation mass spectrometry (ESI-LC–MS).

### Liquid chromatography mass spectrometry

ESI-LC–MS was accomplished using a Dionex UltiMate® 3000 LC system (ThermoScientific), equipped with an UltiMate® 3000 Diode Array Detector (probing 250–400 nm), using Compass 1.3 for esquire HCT Build 581.3, esquireControl version 6.2, Build 62.24 software (Bruker Daltonics), and Bruker compass HyStar 3.2-SR2, HyStar version 3.2, Build 44 software (Bruker Daltonics) at The University York Centre of Excellence in Mass Spectrometry. All mass spectrometry was conducted in negative ion mode. Data analysis was performed using ESI Compass 1.3 DataAnalysis, Version 4.1 software (Bruker Daltonics). A Waters C18 column was used, with a mobile phase of 1% formic acid in water (solvent A) and 0.1% formic acid in acetonitrile (solvent B), used at a flow rate of 0.6 mL min^−1^. The column was pre-equilibrated in solvent A 100% for 1 min, followed by a linear gradient; solvent B 0% to 40% over 5 min. The column was washed in 95% solvent B for 1 min before solvent A 95% was applied for 4 min to re-equilibrate the column.

### Structural prediction

A theoretical coordinate file was generated using phyre2 [52]. Domain architecture was found using the CATH database [53]. A sequence alignment file was generated using COBALT and a structurally informed sequence alignment was generated using ESPript3 and the theoretical structure [54, 55]. A conservation alignment was generated using ConSurf [56–60]. The theoretical structure was aligned with a Protein Data Bank (PDB) file 2GN4 in CCP4MG [61].

### Motility assays

The strains *C. acetobutylicum* ATCC 824 and *C. butyricum* DSM 10702 were grown anaerobically at 37 °C in Reinforced Clostridial Medium (Oxoid™, ThermoScientific™), adjusted to pH 5.4 for *C. acetobutylicum* and pH 7 for *C. butyricum*, with resazurin as an anaerobic indicator (1 mg L^−1^). To detect motility, 5 mL of 2 × YTG medium (16 gL^−1^ tryptone, 10 gL^−1^ yeast extract, 10 gL^−1^ glucose, 5 gL^−1^ NaCl) with 0.4% agar was placed in sterile glass test-tubes. Tubes were stab inoculated with a sterile toothpick using an actively growing culture of each strain and incubated under anaerobic conditions at 37 °C for 48 h. Tubes were photographed to record motility, and readings were made by visually comparing the presence or absence of movement relative to the puncture axis. Motility assays were performed in three independent biological replicates and carried out in a vinyl anaerobic chamber (Coy Laboratory Products).

## Results

### Flagellar glycosylation with Leg and Pse derivatives is predicted to take place in some pathogenic *C. butyricum* strains

**Fig. 1:**
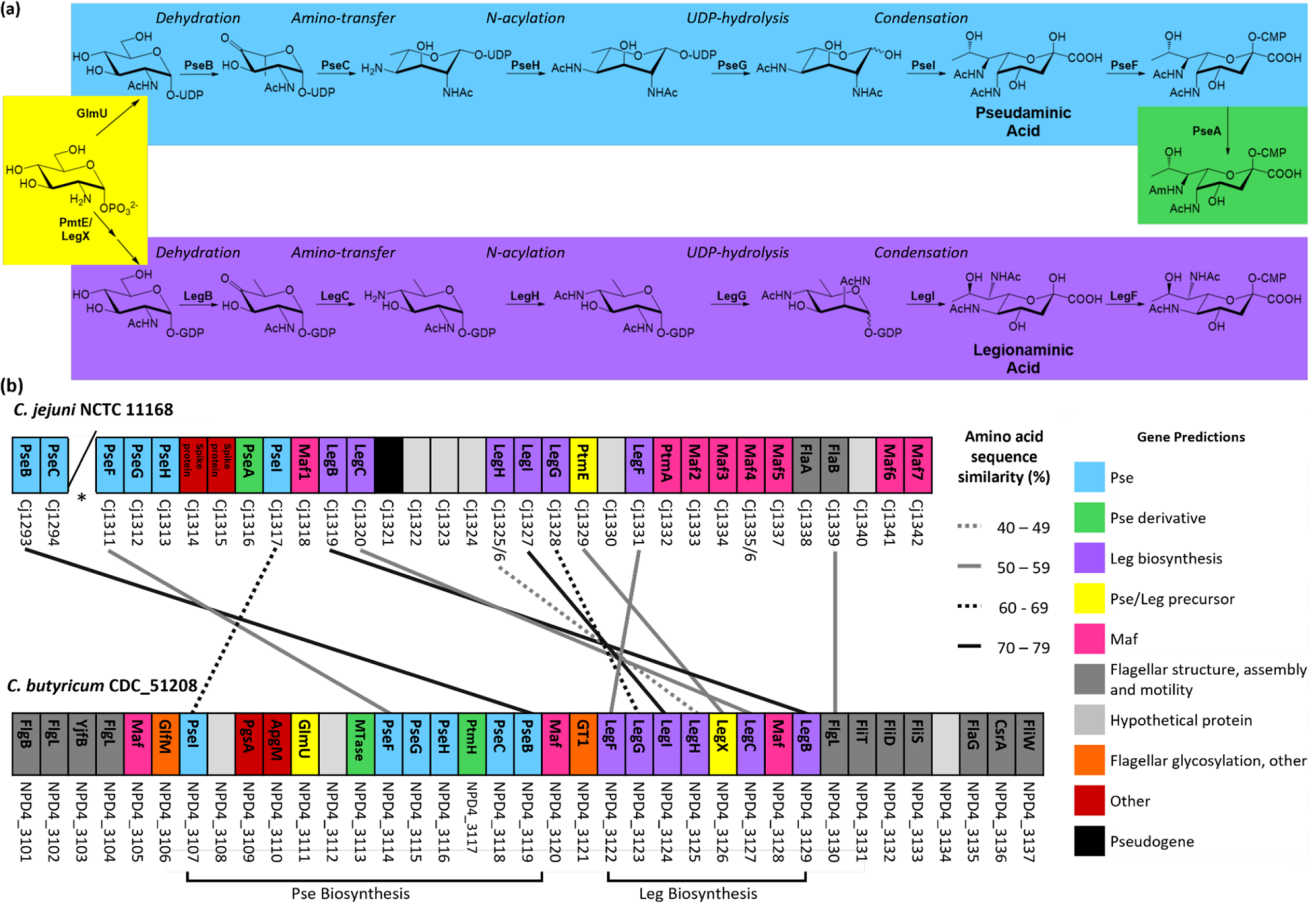
**a** The CMP-Pse (blue) and CMP-Leg (purple) biosynthetic pathways used by *C. jejuni*, and the enzymes required to produce the UDP-GlcNAc and GDP-GlcNAc precursors (yellow) and the CMP-Pse derivative, CMP-Pse5Ac7Am (green). The biosynthesis of Pse and Leg involves five enzymatic steps, namely: dehydration, amino-transfer, *N*-acylation*,* UDP-hydrolysis and condensation. **b** Genes encoded in the flagellar glycosylation locus of *C. jejuni* NCTC 11168 and the conserved cluster of genes found between *flgB* and *fliW* in *C. butyricum* CDC_51208 and several other pathogenic *C. butyricum* strains. *C. butyricum* CDC_51208 orthologues of genes present in the flagellar glycosylation loci of *C. jejuni* NCTC 11168 are indicated with connecting lines, that indicate the level of amino acid sequence similarity. In cases where a *C. jejuni* gene has multiple orthologues in the conserved flagellar locus of *C. butyricum* CDC_51208, that with the highest amino acid sequence similarity has been linked with a line. GlmU, *N*-acetylglucosamine-1-phosphate uridyltransferase; PtmE/LegX, nucleotidyl transferase; PseB, UDP-*N*-acetylglucosamine-5-inverting 4,6-dehydratase; PseC, UDP-2-acetamido-2,6-dideoxy-β-L-arabino-hex-4-ulose aminotransferase; PseH, UDP-4-amino-4,6-dideoxy-β-L-AltNAc *O*-acetyltransferase; PseG, UDP-2,4-diacetamido-2,4, 6-trideoxy-beta-L-altropyranose hydrolase; PseI, pseudaminic acid synthase; PseF, CMP-pseudaminic acid synthetase; PseA, CMP-Pse5Ac7Am acetamidino-synthase; LegB, NAD-dependent GDP-*N*-acetylglucosamine 4,6-dehydratase; LegC, PLP-dependent GDP-4-amino-4,6-dideoxy-α-D-*N*-acetylglucosamine aminotransferase; LegH, GDP-4-amino-4,6-dideoxy-α-D-N-acetylglucosamine *N*-acetyltransferase; LegG, GDP-2,4-diacetamido-2,4,6-trideoxy-α-D-glucopyranose hydrolase/2-epimerase; LegI, legionaminic acid synthase; LegF, CMP-legionaminic acid synthetase; PtmG, CMP-Leg5Am7Ac acetamidino-synthase; PtmH, *N*-methyltransferase; MTase, methyltransferase. *Genes Cj1296–Cj1310 are not involved in flagellar glycosylation and do not have orthologues in the conserved flagellar region of *C. butyricum* CDC_51208

In our previous study, we identified pathogen-specific gene clusters that may be involved in immune invasion and host colonisation [62]. These include a cluster of 37 genes that is situated within the flagellar biosynthesis locus of several pathogenic strains isolated from cases of botulism and necrotising enterocolitis (NEC) (see Table S1, Additional File 1) [62]. In these pathogenic strains, the boundaries of this conserved cluster are marked by the genes *flgB* and *fliW*, which encode the flagellar basal body rod protein and a regulator of flagellar assembly, respectively. Moreover, this cluster encodes a gene annotated as a putative Pse synthase (PseI), an enzyme that catalyses the penultimate step in the biosynthesis of CMP-Pse [63]. As the NulOs Pse and Leg are commonly used to glycosylate flagellin proteins, we used the well-characterised orthologues of the CMP-Pse and CMP-Leg biosynthetic pathways present in *C. jejuni* NCTC 11168, and the putative CMP-Leg biosynthetic genes present in the variable region of the flagellar glycosylation island (FGI-II) of *C. botulinum* F strain Langeland to identify orthologues of these pathways in the botulism-associated strain *C. butyricum* CDC_51208 (Fig. 1; Figure S1; Table S2; Table S3, Additional File 1).

Within the conserved flagellar cluster of *C. butyricum* CDC_51208, we identify clear orthologues (> 50% amino acid sequence similarity) of the genes which encode PseB, PseI and each component of the Leg biosynthetic pathway of *C. jejuni*, with the exception of LegH (Fig. 1; Table S2, Additional File 1). Likewise, we find that 11 of the 18 genes present in the FGI variable region of *C. botulinum* F str. Langeland, which include each of the putative CMP-Leg biosynthetic genes, have orthologues in the flagellar locus of *C. butyricum* CDC_51208 (Fig. 1; Table S3; Figure S1, Additional File 1).

Genes that are predicted to encode the remaining components of the CMP-Pse and CMP-Leg biosynthetic pathways were identified through analysis of their predicted protein domains or their homology to orthologues identified in other species. Genes that encode PseH and LegH, which carry out the *N*-acylation steps in the biosynthesis of CMP-Pse and CMP-Leg, respectively, were identified by the presence of a putative *N*-acetyltransferase domain in their protein sequences (Fig. 1). The genes predicted to encode PseC, PseF and PseG were identified through homology to the *sps* genes of *Bacillus subtilis*, which are involved in the biosynthesis of CMP-Leg and its attachment to the spore surface (Fig. 1) [64]. Furthermore, we identified a gene that encodes an orthologue of the PtmH methyltransferase (MTase), which is required for the biosynthesis of the Leg-derivative Leg5AmNMe7Ac in *Campylobacter coli* VC167 (Fig. 1) [18]. An orthologue of PtmH is also co-encoded with the putative CMP-Leg biosynthetic genes in the FGI variable region of *C. botulinum* F str. Langeland, and is thus likely involved in the biosynthesis of the novel Leg-derivative that is used to decorate its flagella, Leg5GluNMe7Ac (Table S3; Figure S1, Additional File 1) [35]. However, as the PtmH orthologue present in *C. butyricum* CDC_5120 is encoded between the genes *pseH* and *pseC*, we suggest that in this strain it is instead involved in the biosynthesis of a methylated Pse-derivative (Fig. 1). A second, non-homologous methyltransferase is also encoded directly upstream of *pseF* (Fig. 1). Although the exact role of this enzyme is unclear, we find that it is conserved alongside CMP-Pse biosynthesis genes in several related species, and may therefore also be involved in the biosynthesis of a methylated Pse-derivative. Finally, we identified orthologues of *glmU* and *legX*, genes required for the biosynthesis of the CMP-Pse and CMP-Leg precursors, through homology to those previously identified in the species *Leptospira interrogans* (Fig. 1) [65, 66].

In addition to genes that are predicted to encode components of the CMP-Pse and CMP-Leg biosynthetic pathways, we identify genes that encode three non-homologous flagellin proteins within the conserved flagellar cluster (Fig. 1; Table S4, Additional File 1). We also identify genes predicted to encode three motility associated factors (Mafs), the glycosyltransferases that are specifically required for the transfer of NulOs onto flagellin proteins (Fig. 1) [67]. As there is less than 40% amino acid sequence identity between each of these Mafs, we hypothesise that each has a different site specificity or is responsible for the transfer of a different activated NulO derivative onto flagellin (Table S5, Additional File 1). In support of this, Unay et al. (2024) have characterised two Maf paralogs, Maf-1 and Maf-2, which differ in their modes of flagellin binding and serine-target-site specificity [68]. While Maf-1 uses its C-terminal tetratricopeptide repeat (TPR) domain to confer flagellin acceptor and site specificity, Maf-2 requires a co-encoded glycosylation factor for Maf (GlfM) in order to form a ternary complex with flagellin [68]. In *C. butyricum* CDC_51208, we find that only the first Maf (NPD4_3105) is co-encoded with a GlfM orthologue, and that the amino acid sequence identity is greatest between the second (NPD4_3120) and third (NPD4_3128) Mafs encoded in the flagellar cluster (Fig. 1; Table S5, Additional File 1). This suggests that the Maf encoded by NPD4_3105 has a different target-site-specificity to the Mafs encoded by NPD4_3120 and NPD4_3128.

In summary, we predict the presence of genes which encode complete CMP-Pse and CMP-Leg biosynthetic pathways within the flagellar biosynthesis locus of several pathogenic *C. butyricum* strains, exemplified by those present in strain CDC_51208. Moreover, we predict that flagellins of these strains are glycosylated with Leg and a methylated Pse-derivative, through the activity of three Maf proteins.

### Experimental characterisation of *C. butyricum* CDC_51802 PseB as a Pse-biosynthesis enzyme

**Fig. 2:**
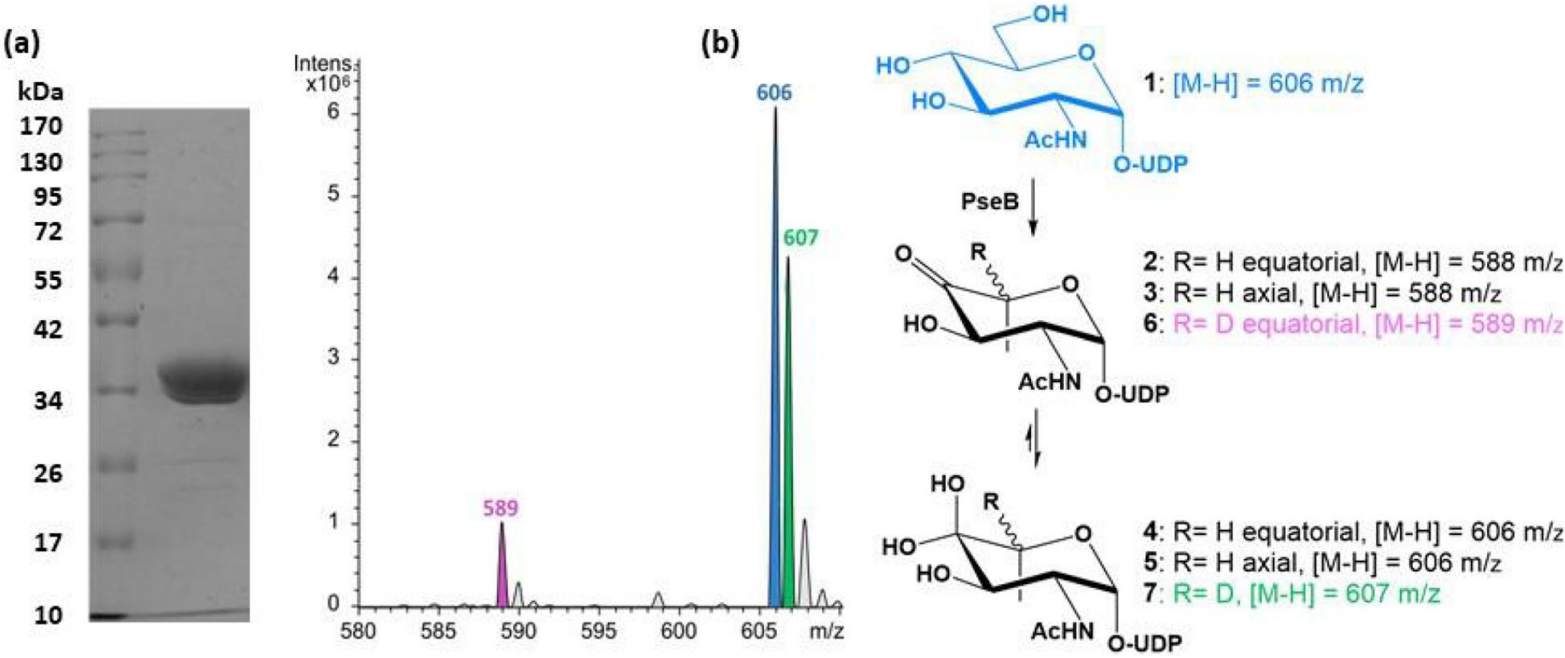
**a** SDS-PAGE analysis of SEC purified PseB (40.3 kDa). **b** LC–MS analysis of PseB activity. Reaction performed in deuterated buffer shows the presence of deuterated enzymatic product UDP-2-acetamido-2,6-dideoxy-β-l-arabino-hexos-4-ulose ([M-H] = 589 m/z)

To test our prediction that *C. butyricum* CDC_51802 encodes orthologues of the Pse biosynthetic pathway, we recombinantly expressed an N-terminally His-tagged version of PseB in *E. coli* Tuner (DE3) cells. Following purification and analysis by SDS-PAGE (Fig. 2a and Additional file 2), the activity of PseB on its substrate (UDP-GlcNAc) 1 (numbered chemical structures are shown in Fig. 2b), was monitored by electron spray ionisation liquid chromatography mass spectrometry (ESI LC–MS). Previously reported characterisation of PseB from *C. jejuni* revealed that the enzyme functions as a 4,6-dehydratase, whilst inverting stereochemistry at C5, to produce UDP-4-keto-6-deoxy-AltNAc 2. PseB can also catalyse a further C5 epimerisation of the initial product to UDP-4-keto-6-deoxy-GlcNAc 3 [69]. However, for this in vitro assay without further downstream enzymatic conversion by PseC, the products of PseB catalysed reactions (2 and 3) exist in equilibrium with hydrated counterparts (4 and 5). Here LC–MS cannot be used to differentiate UDP-GlcNAc 1 starting material from 4 or 5 as each compound is equal in molecular mass ([M − H]^−^ = 606 m*/z*). To combat this, as previously demonstrated for the characterisation of *C. jejuni* PseB, reactions were performed in a deuterated buffer [63]. During the conversion of UDP-GlcNAc to enzymatic product, a non-exchangeable C5-proton from bulk solvent is incorporated, thus performing PseB catalysed reactions in deuterated buffer introduces a C5-deuterium, producing 6 ([M − H]^−^ = 589 m*/z*). Whilst other exchangeable protons of the hydroxyl groups would become deuterated during the assay, they are re-exchanged for protons present in the mobile phase during ESI-LCMS. Therefore, when the enzymatic product is hydrated the resulting deuterated product 7 may be distinguished from UDP-GlcNAc starting material 1, due to a 1 m/z difference. Only 6 and 7 remain deuterated at C5, enabling analysis to distinguish between starting material, enzymatic product and hydrated enzymatic product. LC–MS analysis (Fig. 2b) revealed the presence of untransformed substrate 1, deuterated enzymatic product 6 and the resulting hydrate 7. This confirms that PseB is active as a UDP-*N*-acetylglucosamine 4,6-dehydratase.

### NulO modifications are only one of a number of different glycosylation types predicted in *C. butyricum*

In the next part of our investigation, we examined the content of the flagellar biosynthesis locus in 15 additional complete *C. butyricum* genome assemblies. In line with our previous finding that a cluster of genes which reside between the genes *flgB* and *fliW* are conserved in several pathogenic strains, we determined that across *C. butyricum* strains, the genes *fliD* and *flgB* flank a flagellar HVR that contains between four and 30 genes (Table 1; Table S6, Additional File 1). This flagellar HVR is present in all *C. butyricum* strains except for strain JKY6D1, due to deletion in its entire flagellar biosynthesis locus (Table 1; Table S6, Additional File 1). Moreover, we find that the flagellar HVR of each strain encodes two non-homologous flagellin proteins and FliT, the FliD export chaperone (except for strain CFSA-TJ-E, in which FliT is instead encoded directly upstream of the flagellar HVR) (Table 1; Table S6, Additional File 1). In five of the *C. butyricum* strains, this region incorporates genes that encode putative sugar biosynthesis and flagellin modification enzymes and will therefore be referred to as a flagellar glycosylation island (FGI), a region of DNA that contains genes for the modification of flagella. Moreover, we assigned these *C. butyricum* strains to one of four distinct categories based on the genetic content of their FGIs (Table 1).

**Table 1:** The differences in the size and content of flagellar HVRs across *C. butyricum* strains

Strains	FGI Category	Predicted Flagellin Modification	Genes Encoded by HVR		
			**Total**	**Flagellins**	**Mafs/FGT**
4–1, 16–3, DKU-11, GBW-N1, KNU-L09, LV1, TOA, QXYZ514, CBM588	n/a^a^	None	4 to 5	2	0
CFSA3987, CFSA3989	1	Fuc4N, Glc	13 to 14	2	3 to 4
CBUT	n/a^b^	Methylation	17	2	0
DSM 10702	2	Glc, Par	18	2	3
CFSA-TJ-E	3	Fuc, Gal, Glc, Methylation	19	2	1
CDC_51208	4	Leg, Pse	30	4	3

Table 1 The size of the flagellar HVR in each of the complete *C. butyricum* genome assemblies, in addition to the predicted flagellin modifications and the numbers of flagellins, Mafs, and flagellin glycosyltransferases (FGTs) encoded by the genes in this region*.* Strains have been ordered by the number of genes encoded in the HVR, from smallest to largest. ^a^Flagellar glycosylation genes are absent from the HVR of these strains. ^b^The HVR of strain CBUT is mainly populated by transposase related genes and is not predicted to encode genes involved in flagellar glycosylation

The two strains present in Category 1, CFSA3987 and CFSA3989, which were both isolated from cases of NEC, have FGIs that encode 13 and 14 genes, respectively (Table 1; Table S6, Additional File 1). In addition to *fliT* and the two conserved flagellin genes, the FGIs of these strains encode orthologues of RfbB and RffA, enzymes which sequentially catalyse the biosynthesis of the nucleotide sugar dTDP-fucosamine (dTDP-Fuc4N) from dTDP-Glc (Table S6, Additional File 1). We also identify an enzyme that contains both C-terminal TPR repeats and a predicted glucosyltransferase domain, which is thus likely responsible for the transfer of UDP-glucose onto flagellin (Table S6, Additional File 1). It is therefore predicted that flagellins of strains in Category 1 are decorated with both Fuc4N and glucose.

The *C. butyricum* type strain DSM 10702 (Category 2 FGI), which has 18 genes in its FGI, encodes orthologues of the four components of the CDP-paratose biosynthetic pathway (RfbF, RfbG, RfbH and RfbS) (Table 1; Table S6, Additional File 1) [70, 71]. We also identify a gene predicted to encode a flagellin glucosyltransferase and therefore suggest that flagellins of strain DSM 10702 are glycosylated with glucose and paratose. Encoded within the 19 gene FGI of the botulism-associated strain CFSA-TJ-E (Category 3 FGI) are two GDP-fucose synthetases, a UDP-galactopyranose and the flagellin lysine-*N*-methylase, FliB (Table 1; Table S6, Additional File 1). Also encoded within the FGI is a glycosyltransferase with > 60% amino acid sequence identity to the predicted flagellin glucosyltransferase of strain DSM 10702, in addition to two glycosyltransferase family 2 proteins, which each contain a region homologous to WcaA, a glycosyltransferase responsible for the transfer of UDP-galactose onto a terminal L-fucose residue, during the biosynthesis of colanic acid (Table S6, Additional File 1) [72]. It is therefore predicted that flagellin of strain CFSA-TJ-E is methylated by FliB and decorated with a heteropolymer which comprises glucose, fucose and galactose, and which is linked to flagellin via a glucose residue (Table 1). As discussed in the previous section, it is predicted that the FGI of the botulism-associated strain CDC_51208 (Category 4 FGI) encodes Leg and Pse biosynthetic genes and that flagellins of this strain are glycosylated with both Leg and a methylated Pse-derivative (Table 1). Finally, it was determined that the flagellar HVR of the strain CBUT, which has been prepared for use as a probiotic, contains 17 genes, which include 12 transposase-related genes and *fliB* (Table S6, Additional File 1). Due to the absence of flagellar glycosylation genes from this region, it is predicted that the flagellin of strain CBUT is modified solely by methylation, a process which has previously been shown to enhance host cell adhesion of the enteric bacterium *Salmonella* Typhimurium (Table S6, Additional File 1) [73].

Overall, our analysis of the FGI across *C. butyricum* strains reveals that in general, an increase in length of the FGI results in an increase in the complexity of the predicted flagellin modification (Table 1). We also predict that flagellar glycosylation with both Leg and Pse-derivatives only takes place in pathogenic *C. butyricum* strains.

### A flagellar HVR is a common feature of species of the genus *Clostridium*

After having established the diversity of predicted flagellar modifications in the species *C. butyricum*, we investigated whether a flagellar HVR is also present in other members of the diverse genus *Clostridium*. A total of 98 complete genome assemblies of 39 additional *Clostridium* species were analysed for the presence of a HVR within the flagellar biosynthesis locus (Table S7, Additional File 1).

In line with previous reports that the species *Clostridium baratii* and *Clostridium perfringens* are defective in flagella-mediated motility, we find that flagellar biosynthetic genes are absent from the genomes of these two species (Table S7, Additional File 1) [74, 75]. However, by identifying orthologues of *flgB* and *fliD*, the genes which flank the flagellar HVR in *C. butyricum*, we were able to define a flagellar HVR in the genomes of 33 of the 37 (89%) *Clostridium* species which possess flagellar biosynthesis genes (Table S7, Additional File 1). In the four remaining species: *Clostridium aceticum*, *Clostridium formicaceticum*, *C. kluyveri* and *Clostridium thermosuccinogenes*, the flagellar biosynthetic genes are widely dispersed across the genome and *flgB* and *fliD* are encoded at distant loci, thus precluding the identification of a flagellar HVR (Table S7; Figure S2, Additional File 1). However, as discussed later in this section, we find that FGI-related orthologues are located elsewhere in the genomes of *C. formicaceticum*, *C. kluyveri* and *C. thermosuccigenes*.

In order to determine the most common functions of genes encoded within the flagellar HVRs of *Clostridium* species, we pooled all the genes present in the regions between *flgB* and *fliD* in each of the HVR-containing genomes, and assigned these to orthogroups using OrthoFinder (Additional File 3). We find that the HVR of each *Clostridium* genome contains between two and 89 genes, and that these belong to a total of 274 different orthogroups (Additional File 3). Moreover, we note that the content of the HVR is poorly conserved among species and that no single gene is shared across the HVR of each strain (Additional File 3). However, in 22 of the 33 (66%) *Clostridium* reference genomes which contain a flagellar HVR, we identify at least one Maf-encoding gene, which suggests that the flagellins of these strains are decorated with NulOs (Additional File 3). In line with this, we identify putative NulO biosynthesis genes in the flagellar HVRs of 20 of these reference genomes (Table S8, Additional File 1). These include CMP-Leg biosynthesis genes in the flagellar HVR of *Clostridium manhativorum* CT4, and those responsible for the biosynthesis of an acetamidino-substituted Pse-derivative (Pse5Ac7Am) in the flagellar HVRs of *C. saccharobutylicum*, *C. drakei*, *C. diolis* and *C. novyi* (Table S8, Additional File 1). Although the flagellar HVRs of *Clostridium autoethanogenum* and *Clostridium ljungdahlii* encode Maf proteins, we find that NulO biosynthesis genes are absent from these regions, due to interruptions by transposase and phage sequences. However, in both species we identify a complete set of CMP-Pse biosynthetic genes that are encoded in a different region of the genome, in close proximity to transposase sequences (Table S9, Additional File 1). These CMP-Pse biosynthetic genes may therefore be involved in flagellar glycosylation, although experimental evidence would be required to support this. Likewise, using blastp searches of known CMP-Leg pathway components against all available complete *Clostridium* genomes, we were able to identify CMP-Leg biosynthetic genes that are predicted to be involved in flagellar glycosylation, but are encoded outside the flagellar HVR in the genomes of the species: *Clostridium cellulovorans*, *Clostridium gasigenes* and *Clostridium taeniosporum* (Figure S3, Additional File 1). In each case, CMP-Leg pathway orthologues are encoded alongside Maf proteins, which suggests that CMP-Leg is used for flagellar glycosylation (Figure S2, Additional File 1). In both *C. cellulovorans* and *C. taeniosporum*, the presence of adjacent genes which encode chemotaxis proteins (Clocel_2397 and BGI42_06445, BGI42_06450, BGI42_06600, respectively) further supports this hypothesis. It was also noted that while the flagellar HVR of *C. taeniosporum* 1/k is predicted to be composed of CMP-Pse biosynthesis genes, the flagellar HVRs of *C. cellulovorans* 743B and *C. gasigenes* CGAS001 do not contain genes that are predicted to be involved in flagellar glycosylation (Table S7, Additional File 1; Additional File 3). Finally, we determine the widespread presence of genes that encode methylases, acetyltransferases and glycosyltransferases in the FGIs of *Clostridium* strains (Additional File 3). While the specificity of these enzymes could not be determined, it is likely that these are involved in adding to the variety and complexity of the flagellin glycan structures.

**Fig. 3:**
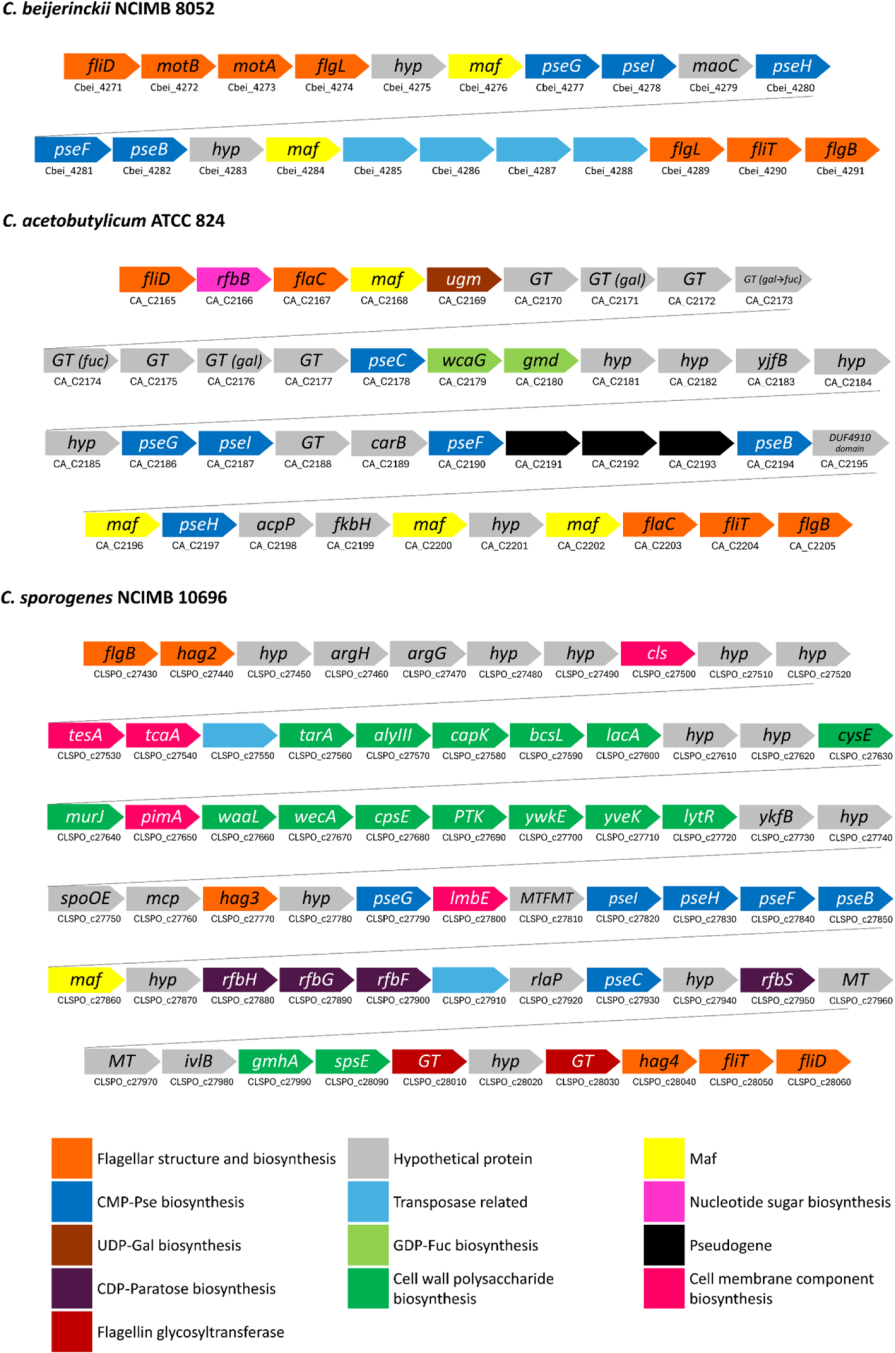
The organisation of genes in the FGIs of *C. beijerinckii* NCIMB 8052, *C. acetobutylicum* ATCC 824 and *C. sporogenes* NCIMB 10696, flanked by the flagellar structural genes *fliD* and *flgB*

To exemplify the presence of genes that are most frequently found in the FGIs of *Clostridium* species, we focussed on those present in the FGIs of the industrially relevant species *C. acetobutylicum* and *C. beijerinckii*, in addition to the gut commensal *Clostridium sporogenes* (Fig. 3).

Within the FGI of the *C. beijerinckii* type strain NCIMB 8052, we identified genes that encode two Mafs, two flagellin proteins and orthologues of each component of the CMP-Pse biosynthetic pathway, except for PseC (Fig. 3). Also encoded in the FGI of *C. beijerinckii* NCIMB 8052 is the flagellin methyltransferase FliB, the motility proteins MotA and MotB, and four transposase-related genes (Fig. 3). Moreover, we predict that the gene Cbei_4882, which is located at a separate locus in the genome, encodes an orthologue of PseC. Therefore, we predict that the flagellin of *C. beijerinckii* NCIMB 8052 is both methylated and decorated with Pse.

Like *C. beijerinckii*, we determined that the FGI of the industrial solvent-producing strain *C. acetobutylicum* ATCC 824 encodes two flagellins and orthologues of each component of the CMP-Pse biosynthetic pathway (Fig. 3). However, also encoded within the FGI are genes predicted to be involved in the biosynthesis of the nucleotide-linked sugars UDP-galactopyranose and GDP-fucose (Fig. 3). In line with this, we identify genes that are predicted to encode three galactosyltransferases and a fucosyltransferase, through their homology to the glycosyltransferases WcaA and WcaE, respectively (Fig. 3) [49]. Although we were unable to predict the specificity of the five other glycosyltransferases encoded within the FGI of this strain, we determined that these are unlikely to serve as FGTs, due to the absence of TPR repeats from their amino acid sequences. However, we were able to identify four Maf genes, via homology to those present in *C. jejuni* (Fig. 3). Overall, this suggests that flagellin of *C. acetobutylicum* ATCC 824 is decorated with Pse and a glycan that contains both galactose and fucose monomers.

Finally, we predict that the FGI of *C. sporogenes* NCIMB 10696 encodes each of the components of the CMP-Pse biosynthetic pathway and genes required for the biosynthesis of a 3,6-dideoxyhexose sugar, in addition to those involved in the biosynthesis of cell wall polysaccharides and components of the cell membrane (Fig. 3). The cell envelope-related genes are encoded between the first and second flagellin genes present in the FGI, while those predicted to be involved in flagellar glycosylation are situated between the second and third flagellin encoding genes (Fig. 3). In addition to a singular *maf* gene, we identify genes that are predicted to encode two possible FGTs, based on the presence of GT2 domains with TPR repeats in their amino acid sequences, although these would differ from canonical Maf proteins (Fig. 3). We therefore predict that the flagellin of *C. sporogenes* NCIMB 10696 might be glycosylated with both Pse and a 3,6-dideoxyhexose sugar.

For completion, we returned our attention to the four *Clostridium* species that contain flagellar biosynthesis genes, but which do not contain a flagellar HVR due to the unusual dispersal of flagellar biosynthetic genes across the genome (Figure S2, Additional File 1). We searched the entire genomes of each of these species for genes which may be involved in flagellar glycosylation. While the genome of *C. aceticum* does not appear to contain genes involved in flagellar glycosylation, we determined that genes that are predicted to be involved in NulO and nucleotide sugar biosynthesis are encoded in close proximity to Maf or flagellin proteins in the reference genomes of *C. formicaceticum*, *C. kluyveri* and *C. thermosuccinogenes*, and are thus likely to be involved in flagellar glycosylation in these species (Table S10, Additional File 1). We identify orthologues of the components of the complete CMP-Leg/Pse and CMP-Leg biosynthetic pathways in the genomes of *C. formicaceticum* DSM 92 and *C. kluyveri* NBRC 12016, respectively, while genes predicted to be involved in flagellar glycosylation with a glycan that contains glucose and a 3,6-dideoxyhexose sugar are located between *flgL* and *fliD* in the genome of *C. thermosuccinogenes* DSM 5807 (Table S10).

In the last part of our analysis we note that, in addition to genes predicted to be involved in flagellar glycosylation, 15 of the 274 orthogroups (5%) identified in the pooled flagellar HVR genes of *Clostridium* strains contain transposase-related genes (Additional File 3). Likewise, the highly diverse contents of these FGIs and the presence of inserts of other gene functions suggest that the variation observed in this region of the genome has evolved via the introduction of genes during transposition events (Additional File 3). Furthermore, in several cases where we identify putative flagellar glycosylation genes at genomic loci outside the flagellar HVR, we find that these are encoded in close proximity to transposase sequences, endonucleases and prophage elements, which is suggestive of horizontal gene transfer (Figure S3; Table S10, Additional File 1).

### Flagellar glycosylation with NulOs is predicted to occur in both pathogenic and non-pathogenic *Clostridium* species

To date, reports of flagellar glycosylation with NulOs have largely been restricted to pathogenic species/strains of bacteria. Of the 15 available complete *C. butyricum* genome assemblies that contain flagellar HVRs, we find that NulO biosynthetic genes are only present in the pathogenic strain CDC_51208. Furthermore, in our previous analysis, we determine that the content of this region is conserved in several other NEC and botulism-associated strains with incomplete genome assemblies (Table S6, Additional File 1) [62]. However, tblastn searches for each of the genes predicted to be involved in CMP-Pse and CMP-Leg biosynthesis in strain CDC_51208 against all available incompletely assembled *C. butyricum* genomes revealed that, in addition to their presence in strains isolated from cases of botulism and NEC, a complete set of CMP-Leg biosynthetic genes are encoded in the FGIs of several *C. butyricum* strains isolated from soil, the faecal sample of a preterm infant and the gut microbiome of other animal species (Table S11, Additional File 1).

**Fig. 4:**
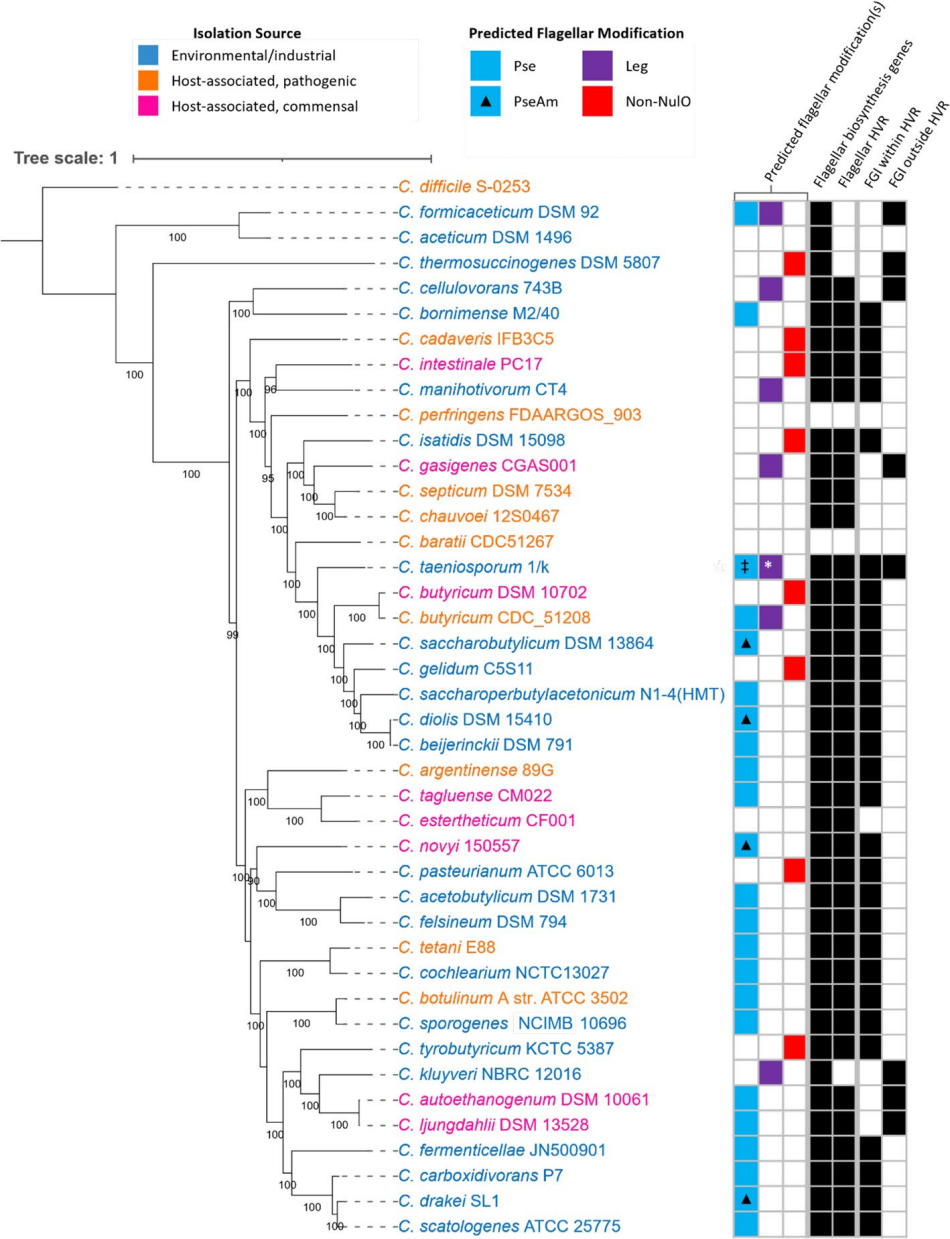
The phylogenetic relationships of two *C. butyricum* strains (DSM 10702 and CDC_51208) and 39 other *Clostridium* species which have complete genome assemblies, in addition to the isolation source of each reference strain, and the nature of the flagellar modifications that are predicted to be encoded by genes present in their FGIs. *C. difficile* S-0253 was used as the outgroup, and the phylogenetic tree was constructed by a method of maximum likelihood based on the concatenation of 232 core genes. Numbers at the tree nodes represent bootstrap support values > 90% (1,000 replications). Black cells indicate the presence of the listed feature. ‡Pse modification is encoded within the flagellar HVR. *Leg modification is encoded outside the flagellar HVR

Looking across the whole of the genus, we find that there is no correlation between the environment from which the species were isolated and the presence of NulO biosynthetic genes, and that NulO biosynthesis genes are not restricted to pathogens, as is largely observed for Gram-negatives species (Fig. 4; Table S8, Additional File 1) [6]. In addition, the distribution of the *Clostridium* species that contain NulO biosynthetic genes does not correlate with their organismal phylogeny (Fig. 4). As Pse biosynthetic genes that are predicted to be involved in flagellar glycosylation are present in 20 of the 37 flagellated *Clostridium* species, we also examined the phylogeny of PseI as an example protein, but find that this does not correlate with organismal phylogeny (Figure S4, Additional file 1). Therefore, it is parsimonious that the patchy distribution of NulO biosynthetic genes across the genus has occurred through extensive horizontal gene transfer, which is consistent with the identification of transposase related genes in the FGIs of many of these species.

## Discussion

In this study, we report the widespread occurrence of a flagellar HVR across the bacterial genus *Clostridium*. We have determined that this HVR resides between the flagellar structural genes *flgB* and *fliD* and predict its involvement in flagellar glycosylation. In *C. butyricum*, the type species of the genus *Clostridium*, we identify a flagellar HVR in 15 of the 16 available complete genome assemblies. In five of these strains, which include the non-pathogenic strain DSM 10702 and four strains isolated from cases of botulism and NEC, this HVR contains genes which encode sugar biosynthesis genes and glycosyltransferases. We predict that the strain DSM 10702 decorates its flagellins with both glucose and the 3,6-dideoxyhexose sugar paratose, and that glucose is also present in the flagellar glycans of the three pathogenic strains: CFSA3987, CFSA3989 and CFSA-TJ-E. In support of the presence of glucose in the flagellar modifications of these strains, it has previously been reported that d-glucose and GlcNAc are incorporated in the flagellar glycans of *C. tyrobutyricum* ATCC 25755, in a ratio of 11:1 [40]. Likewise, orthologues of the genes involved in the biosynthesis of a CDP-paratose precursor, CDP-4-keto-6-deoxy-d-glucose, have been identified in the FGIs of several non-pathogenic species of the genera *Erwinia* and *Pantoea*, which supports our prediction that a paratose-like sugar is involved in flagellar glycosylation in *C. butyricum* [76]. In addition to glucose, we predict that Fuc4N and both fucose and galactose are also present in the flagellar glycans of strains CFSA3987/CFSA3989 and CFSA-TJ-E, respectively. It is notable that the sugars fucose and galactose form a major component of the glycans which decorate host colonic mucin and therefore may play a role in immune evasion [77]. Likewise, the dTDP-6-deoxy-hex-4-ulose derivative Fuc4N, although not previously reported to be present in flagellar glycans, is a component of the cell surface enterobacterial common antigensurface polysaccharide present in members of the family Enterobacteriaceae, in which has been proposed to function in host–pathogen interactions [78]. Furthermore, it has been reported that genes involved in the biosynthesis of a dTDP-6-deoxy-hex-4-ulose derivative are encoded in the FGI of a *Pectobacterium* species, which supports our prediction that a Fuc4N derivative is present in the flagellar glycan of these three *C. butyricum* strains [76]. Finally, we find that complete sets of genes required for the biosynthesis of the NulOs CMP-Pse and CMP-Leg, which are used for flagellar glycosylation in the major gut pathogens *H. pylori* and *C. jejuni*, are only present in the botulism-associated strain CDC_51208 [3, 11]. This cluster of genes is also conserved in several other botulism and NEC-associated strains that have incomplete genome assemblies, which is suggestive of a role in host colonisation.

Before we discuss the presence of NulOs in these pathogenic strains, we note that a complete set of CMP-Leg biosynthetic genes were also identified in the flagellar HVR of several incomplete genome assemblies for *C. butyricum* strains isolated from soil, the gut microbiome of other animal species and a preterm infant. Although these strains were not specifically isolated from cases of infection, it has been established that the intestinal tracts of other animals act as disease reservoirs, and that pathogenic Clostridia frequently reside in the soil environment [79–85]. Furthermore, it has been determined that the first colonising strains of preterm infants are often hospital-associated pathogens [86, 87]. Therefore, we suggest that *C. butyricum* strains that have been isolated from these niches and which glycosylate their flagellins with Leg may have the propensity to cause disease in a human host. In line with this, it has been determined that a strain of the species *Enterococcus faecium*, which primarily exists as gut commensal, but can also act as an opportunistic pathogen, expresses a Leg-containing cell surface glycan [88]. Likewise, it has been suggested that NulO biosynthetic genes found in environmental isolates of the species *Vibrio vulnificus* may function during opportunistic pathogenesis of host species [89]. Alternatively, it is possible that flagellar glycosylation with Leg may be involved in host colonisation in commensal *C. butyricum* strains. In support of this, it has previously been determined that several isolates of the principal human gut–associated methanogen, *Methanobrevibacter smithii*, decorate their cell surface with Pse in order to mimic host sialic acids present in the intestinal niche [90, 91].

The first possible role of flagellar glycosylation with NulOs in pathogenic *C. butyricum* strains is in flagellar assembly. In the species *C. jejuni*, *H. pylori* and *A. caviae*, it has been established that the glycosylation of flagellin with NulOs is required for flagellar assembly and thus motility, which facilitates host colonisation [10, 11, 92]. However, we suggest that in *C. butyricum*, at least, flagellar glycosylation with NulOs could serve another purpose, due to observations that the strain DSM 10702 is motile, despite our prediction that its flagellins are not glycosylated with NulOs, but rather with other sugars (Figure S5). We therefore suggest that in pathogenic *C. butyricum* strains, flagellar glycosylation with NulOs may instead facilitate host colonisation through immune evasion, due to the structural similarity of Pse and Leg to the sialic acids present at the termini of host colonic mucin glycans [77]. In line with this, it has been determined that the host innate immune system is less able to detect flagellins decorated with NulOs as foreign [93]. Specifically, it has been determined that during host colonisation, bacteria that use sialic acid mimicry can exploit host factor H and/or Siglec-9 and Siglec-10 to down-regulate the host complement pathway and dampen the response of neutrophils [93–96]. Furthermore, our findings that flagellar glycosylation with NulOs generally takes place in pathogenic *C. butyricum* strains are reflected in the study of Twine et al. (2008), in which it was predicted that the flagellins of three of the four strains isolated from infant botulism cases are decorated with Leg-derivatives, while the flagellins in three of the four *C. botulinum* strains which are not associated with botulism cases are instead glycosylated with di-*N*-acetylhexuronic acid derivative moieties [35]. In a further study in which microarray analysis of the coding sequences present in the FGI of 58 *C. botulinum* strains was carried out, it was revealed that strains fall into six divisions which may confer host specificity and disease pathotype [35, 36]. It has also been suggested that the heterogeneity observed across the flagellar glycans of *C. jejuni* strains is likely to allow optimal interaction with different hosts [6]. This is exemplified by a set of six genes (*cj1321*-*cj1326*) that are required for the biosynthesis of Leg, and which are conserved in the flagellar glycosylation locus of livestock-associated strains [5, 97]. Furthermore, phase variation of Leg and Pse biosynthetic genes may be utilised as an immune evasion strategy, as has been observed in *C. jejuni* [98, 99].

Overall, we speculate that the predominance of flagellar glycosylation in pathogenic *C. butyricum* strains and the incorporation of sugars that are involved in virulence in other pathogenic species into the flagellar glycans of these strains suggests that primary role of flagellar glycosylation in the species *C. butyricum* is in immune evasion and host colonisation. However, further experimental evidence will be required to support these suggestions.

In the second part of our study, we expand our investigation across the genus *Clostridium*. Building on previous observations that most *Clostridium* species are motile, we determine that a flagellar HVR is present in 89% of *Clostridium* species that possess flagellar biosynthesis genes [100]. Moreover, we identify NulO biosynthesis genes in over half of these species, which include those isolated from a range of niches. In Gram-negative bacteria, reports for flagellar glycosylation with NulOs have predominantly been for pathogenic species [6]. However, the prevalence of NulO biosynthesis genes in the FGIs of both pathogenic and non-pathogenic *Clostridium* species suggests that flagellar glycosylation with NulOs serves a range of functions, beyond immune evasion. In support of this, there have been several reports of the presence of NulOs on the cell surface of environmentally isolated non-pathogenic bacteria such as the species *Candidatus* Accumulibacter, which produces an extracellular matrix that contains Pse and Leg, and that NulO biosynthetic pathways are present in environmental isolates of the non-pathogenic species *Flavobacterium johnsoniae*, *Leeuwenhoekiella blandensis* and *Idiomarina loihiensis* [91, 101]. Moreover, Kleikamp et al*.* (2020), using mass spectrometry, applied a large-scale survey approach to determine that NulOs are frequently found in non-pathogenic prokaryotic species isolated from a variety of environmental niches [102].

First, it is likely that in several *Clostridium* species, flagellar glycosylation with NulOs is required for bacterial motility. It has been well established that in many Gram-negative pathogens (e.g. *C. jejuni*, *H. pylori* and *A. caviae*) and the Gram-positive pathogens *C. difficile* and *Paenibacillus alvei*, the glycosylation of flagellin with NulOs is required for flagellar filament assembly and thus motility [10, 11, 32, 92, 103–105]. Moreover, a previous comparative genome analysis of 11 *C. tetani* strains revealed that the only strain with a complete Pse biosynthesis pathway was also the only strain to show significant swarming motility on blood agar [38]. It has therefore been suggested that flagellar glycosylation with Pse is required for motility in this species [38]. It has also been postulated that flagellar glycosylation with NulOs is involved in the motility of the non-pathogenic species *C. acetobutylicum* [41]. Through our analysis of the genetic content of *C. acetobutylicum* ATCC 824, we predict that this strain decorates its flagellin with Pse. In line with this, it was determined that this strain is modified with a terminal sialyl-like residue following cleavage using the neuraminidase from *Arthrobacter ureafaciens*, which potentially demonstrates pseudaminidase-like promiscuity, although such activity remains to be confirmed [41, 106]. Furthermore, it was demonstrated that a ~ 10 kDa modification remains on the flagellin protein after neuraminidase treatment [41]. This supports our prediction that the flagellin of this strain is also glycosylated with a heteropolymer that contains both fucose and galactose residues. Moreover, in a previous study, it was determined that despite having identical flagellin amino acid sequences, a non-motile mutant produced flagellin with a molecular weight of 30 kDa compared to the wild-type molecular weight of ~ 43 kDa [107]. This weight discrepancy aligns with that of the flagellin modification determined by Lyristis et al*.* (2000), and it has therefore been suggested that flagellar glycosylation may allow flagellin to fold into a highly motile conformation to allow chemotaxis towards fermentable sugars, and to maintain flagellar stability under acidic conditions [41, 108].

Further physical benefits imparted by NulOs-mediated flagellar glycosylation in non-pathogenic *Clostridium* species may include strengthening and protection of the flagellum. In *C. jejuni*, it has been proposed that glycosylation with Pse and its derivative Pse5Ac7Am, which we predict is also used for flagellar glycosylation in the species *C. saccharobutylicum*, *C. drakei*, *C. diolis* and *C. novyi*, strengthens the flagellum [18]. Likewise, although the flagellins of *Pseudomonas syringae* are instead glycosylated with a rhamnose and viosamine containing trisaccharide, it has been experimentally demonstrated that this allows optimal motility by stabilising the flagellar filament and aiding its rotation [109, 110]. For both *C. coli* and *C. acetobutylicum*, it has been suggested that flagellar glycosylation with NulOs may provide resistance to degradation by proteases secreted by other species of the gut and soil microbiomes [41, 111]. The presence of flagellar NulOs is likely to contribute to the formation of a repellent protective barrier around the bacterial cell, due to their negatively charged nature.

In pathogenic *Clostridium* species, in addition to a role in host immune evasion as discussed for *C. butyricum*, flagellar glycosylation with NulOs may have a role in biofilm formation and host colonisation. Howard et al*.* (2009) previously determined that *C. jejuni* mutants which were unable to express Leg were significantly less able to form biofilms [5]. Similarly, it was found that *C. jejuni* 81–176 and *C. coli* VC167, for which autoagglutination is involved in biofilm formation, mutants unable to produce PseAm or PseAc flagellar modifications failed to auto-agglutinate and showed attenuated virulence in a ferret model [9]. It has also been proposed that the negatively charged regions present in the type B flagellin modification of hypervirulent *C. difficile* strains facilitate ionic interactions between the flagella and extracellular structures during host colonisation [105]. Valiente et al. (2016) further demonstrated that type B flagellin modification of *C. difficile* facilitates adhesion to intestinal epithelial cells and is involved in biofilm formation on abiotic surfaces [112].

Finally, we suggest that across the genus *Clostridium*, the region of the genome between the genes *flgB* and *fliD* serves as a hotspot for the integration of foreign DNA. Within this region, we identify hallmarks of horizontal gene transfer, which include transposase sequences and inserts of a range of gene functions, which are unrelated to flagellar biosynthesis or modification. Moreover, in many cases where NulO biosynthesis genes are present in the genome at loci outside the flagellar biosynthesis locus, we find that these are flanked by transposases, prophage elements and endonuclease sequences. In support of these findings, it has previously been determined that the FGI present in *C. botulinum* strains has evolved independently from the remainder of the genome, and that the CMP-Pse biosynthesis gene-encoding HVR of *C. tetani* strains is flanked by mobile genetic elements [36, 38]. Evidence of horizontal gene transfer has also been identified in the FGIs of several Gram-negative species, which include *P. aeruginosa* and those of the family Enterobacteriaceae [33, 113]. In *P. aeruginosa*, it has been suggested that the FGI has been acquired via horizontal gene transfer via recombination sites in the flagellar genes *fleP* and *flgJ* and that transposase sequences are ubiquitous in enterobacterial FGIs [76, 113].

## Conclusions

In summary, we find that a flagellar HVR, which varies considerably in size and content across strains and species, is a common feature of the genus *Clostridium*. We predict that this region is responsible for the modification of flagellin with a wide variety of glycans, thus expanding the previously limited reports of flagellar glycosylation in Gram-positive species. In *C. butyricum*, the type species of the genus, we predict the presence of several novel flagellar glycans and find that flagellar glycosylation with NulOs primarily takes place in pathogenic strains. Although previous reports for flagellar glycosylation with NulOs have primarily been for pathogenic Gram-negative species, we predict that the modification of flagellin with NulOs is widespread in both pathogenic and non-pathogenic members of the genus *Clostridium*. These findings have potential applications in the identification and characterisation of pathogenic strains and in the design of strains engineered for use in both industrial biotechnology and as novel therapeutics.

## Supplementary Information

Additional file 1. Supplementary information

Additional file 2. Original SDS-PAGE gel image for SEC purified PseB

Additional file 3. *Clostridium *flagellar HVR orthologs table. This Excel workbook contains a table of OrthoFinder data for each of the 33 *Clostridium *species that possess a flagellar HVR.

## Data Availability

All data generated or analysed during this study are included in this published article and its supplementary information files.

## Abbreviations

AT: Acetyltransferase
BLAST: Basic Local Alignment Search Tool
CDP: Cytidine diphosphate
CMP: Cytidine monophosphate
dTDP: Deoxythymidine diphosphate
ESI-LC-MS: Liquid chromatography-electrospray ionisation mass spectrometry
FGI: Flagellar glycosylation island
FGT: Flagellin glycosyltransferase
Fuc4N: Fucosamine
GDP: Guanosine diphosphate
GlcN: Glucosamine
GlcNAc: *N*-Acetylglucosamine
GlfM: Glycosylation factor for Maf
HPLC: High performance liquid chromatography
HVR: Hypervariable region
Leg: Legionaminic acid
Maf: Motility associated factor
MTase: Methyltransferase
NADPH: Nicotinamide adenine dinucleotide phosphate
NAT: *N*-Acetyltransferase
NCBI: National Centre for Biotechnology Information
NEC: Necrotising enterocolitis
Neu5Ac: *N*-Acetylneuraminic acid
NTP: Nucleotidyltransferase
NulO: Nonulosonic acid
PLP: Pyridoxal phosphate
Pse: Pseudaminic acid
SEC: Size exclusion chromatography
TPR: Tetratricopeptide repeat
UDP: Uridine diphosphate
