# Supplementary material for: Flagellar glycosylation with pseudaminic acids is widespread in the genus Clostridium
Source: BMC Microbiol. 2026 May 6;26:574. doi: 10.1186/s12866-026-04975-z (PMC13317340; doi:10.1186/s12866-026-04975-z)
Supplement: Supplementary file 1 — Additional file 1. Supplementary information [file 12866_2026_4975_MOESM1_ESM.pdf]

Supplementary Material

**Table S1**	…………………………………………………………………………………..	**2**
**Table S2**	…………………………………………………………………………………..	**3**
**Table S3**	…………………………………………………………………………………..	**5**
**Figure S1**	…………………………………………………………………………………..	**7**
**Table S4**	…………………………………………………………………………………..	**8**
**Table S5**	…………………………………………………………………………………..	**9**
**Table S6**	…………………………………………………………………………………..	**10**
**Table S7**	…………………………………………………………………………………..	**14**
**Figure S2**	…………………………………………………………………………………..	**18**
**Table S8**	…………………………………………………………………………………..	**19**
**Table S9**	…………………………………………………………………………………..	**20**
**Figure S3**	…………………………………………………………………………………..	**21**
**Table S10**	…………………………………………………………………………………..	**22**
**Table S11**	…………………………………………………………………………………..	**24**
**Figure S4**	…………………………………………………………………………………..	**26**
**Figure S5**	…………………………………………………………………………………..	**27**
**References**	…………………………………………………………………………………..	**28**

Table S1. Pathogenic *C. butyricum* strains that contain a conserved cluster of genes within the flagellar biosynthesis locus, and the locus tags of *flgB* and *fliW*, the two genes which flank this cluster in each strain.

C. butyricum **Strain**	**GenBank Accession**	**Reference**	**Conserved Region**	
			flgB	***fliW***
300064	GCA_002940805.1		AWN73_RS10640	AWN73_RS10450
5521	GCA_000171115.1	(1)	CBY_3585	CBY_3622
ATCC 43755	GCA_011017415.1		FDB92_RS09195	FDB92_RS10555
BL-5262-9RE	GCA_011019995.1		FDE75_RS09100	FDE75_RS08920
BoNT BL5262	GCA_000182605.1		CLP_RS04640	CLP_RS04815
CDC_51208	GCA_001886875.1	(2)	NPD4_3101	NPD4_3137
NOR 33234	GCA_000785185.1	(3)	OA81_RS08075	OA81_RS08280

Table S2. Locus tags and predicted functions of the genes encoded in the flagellar glycosylation locus of *C. jejuni* NCTC 11168 and their top BLASTP hits in the genome of *C. butyricum* CDC_51208. Gene products which have been established as components of the CMP-Pse and CMP-Leg biosynthetic pathways have been highlighted in bold.

***C. jejuni* NCTC 11168**		***C. butyricum* CDC_51208**		
**Locus Tag**	**Predicted Function**	**Top BLASTP Hit**	**Amino Acid Sequence Similarity (%)**	**Query Cover (%)**
Cj1293	**PseB**	NPD4_3119	70	98
Cj1294	**PseC**	NPD4_2779[a]	52	98
Cj1295	Conserved hypothetical protein	-	-	-
Cj1296	Hypothetical protein	-	-	-
Cj1297	Hypothetical protein	-	-	-
Cj1298	Aminoglycoside N(3)-acetyltransferase	-	-	-
Cj1299	Putative acyl carrier protein	-	-	-
Cj1300	Putative SAM domain containing methyltransferase	-	-	-
Cj1301	Hypothetical protein	-	-	-
Cj1302	Putative HAD-superfamily phosphatase	-	-	-
Cj1303	Putative 3-oxoacyl-[acyl-carrier-protein] synthase	-	-	-
Cj1304	Putative acyl carrier protein	-	-	-
Cj1305	Hypothetical protein	-	-	-
Cj1306	Hypothetical protein	-	-	-
Cj1307	Putative amino acid activating enzyme	NPD4_3176[a]	49	93
Cj1308	Putative acyl carrier protein	-	-	-
Cj1309	Hypothetical protein	-	-	-
Cj1310	Hypothetical protein			
Cj1311	**PseF**	NPD4_3122	51	87
Cj1312	**PseG**	-	-	-
Cj1313	**PseH**	-	-	-
Cj1314	Imidazole glycerol phosphate synthase subunit	NPD4_2782[a]	56	100
Cj1315	Imidazole glycerol phosphate synthase subunit	NPD4_2784[a]	65	100
Cj1316	PseA	-	-	-
Cj1317	**PseI**	NPD4_3107	65	96
Cj1318	Maf1	NPD4_3128	42	34
Cj1319	**LegB**	NPD4_3129	72	100
Cj1320	**LegC**	NPD4_3127	58	94
Cj1321	Putative transferase	-	-	-
Cj1322	Hypothetical protein	-	-	-
Cj1323	Hypothetical protein	-	-	-

Cj1324	Hypothetical protein	-	-	-
Cj1325/6	**LegH**	NPD4_3117	43	69
Cj1327	**LegI**	NPD4_3124	74	99
Cj1328	**LegG**	NPD4_3123	67	99
Cj1329	Putative sugar-phosphate nucleotide transferase	NPD4_3126	57	96
Cj1330	Hypothetical protein	-	-	-
Cj1331	**LegF**	NPD4_3122	54	95
Cj1332	PtmA	-	-	-
Cj1333	Maf2[b]	NPD4_3128	44	39
Cj1334	Maf3	NPD4_3120	40	62
Cj1335/6	Maf4[c]	NPD4_3128	42	34
Cj1337	Maf5	NPD4_3128	41	49
Cj1338	FlaB	NPD4_3130	55	34
Cj1339	FlaA	NPD4_3130	54	49
Cj1340	Conserved hypothetical protein	NPD4_3120	44	76
Cj1341	Maf6	NPD4_3120	46	77
Cj1342	Maf7	NPD4_3120	44	75

[a]Gene is not encoded within the conserved flagellar glycosylation cluster of *C. butyricum* CDC_51208. [b]Maf2 is CMP-PseAm specific. [c]Maf4 is CMP-Leg specific.

Table S3. Locus tags and predicted functions of the genes encoded in the FGI of *C. botulinum* F str. Langeland, and their orthologs in the genome of *C. butyricum* CDC_51208. Gene products that are putatively involved in the biosynthesis of CMP-Leg have been highlighted in bold.

***C. botulinum* str. F Langeland**		***C. butyricum* CDC_51208**		
**Locus Tag**	**Predicted Function**	**Top BLASTP Hit**	**Amino Acid Sequence Similarity (%)**	**Query Cover (%)**
Flagellar Glycosylation Island Conserved Region (FGI-I)[a]				
CLI_2735	Argininosuccinate lyase	NPD4_1027[b]	73	100
CLI_2736	Argininosuccinate synthase	NPD4_1028[b]	76	98
CLI_2737	Conserved hypothetical protein	-	-	-
CLI_2738	Conserved hypothetical protein	-	-	-
CLI_2739	Cardiolipin synthetase	NPD4_935[b]	61	98
CLI_2740	Putative membrane protein	NPD4_307[b]	65	52
CLI_2741	Conserved hypothetical protein	-	-	-
CLI_2742	VanZ family protein	NPD4_598[b]	60	91
CLI_2743	Polysaccharide biosynthesis protein	-	-	-
CLI_2744	NAD-dependent epimerase/dehydratase family protein	NPD4_1075[b]	55	97
CLI_2745	NeuB family protein	NPD4_3124	56	95
CLI_2746	Cytidylyltransferase domain protein	NPD4_3115	50	44
CLI_2747	Putative membrane protein	-	-	-
CLI_2748	Exopolysaccharide biosynthesis protein	-	-	-
CLI_2749	Exopolysaccharide biosynthesis protein	-	-	-
CLI_2750	UDP-galactopyranose mutase	-	-	-
CLI_2751	Putative undecaprenyl-phosphate galactosephosphotransferase	-	-	-
CLI_2752	Capsular exopolysaccharide family protein	NPD4_779[b]	69	98
CLI_2753	Exopolysaccharide biosynthesis protein	NPD4_780[b]	64	99
CLI_2754	Capsular polysaccharide biosynthesis protein	NPD4_778[b]	63	98
CLI_2755	Cell envelope-related transcriptional attenuator domain protein	NPD4_2576[b]	67	80

CLI_2756	Conserved hypothetical protein	NPD4_3103	63	98
CLI_2757	Hypothetical protein	-	-	-
CLI_2758	Conserved hypothetical protein	-	-	-
CLI_2759	Methyl-accepting chemotaxis protein	NPD4_1677[b]	53	95
CLI_2760	Flagellin	NPD4_3130	58	92
CLI_2763	Pseudogene	-	-	-

Flagellar Glycosylation Island Variable Region (FGI-II)[a]

CLI_2764	Conserved hypothetical protein	-	-	-
CLI_2765	Hypothetical protein	-	-	-
CLI_2766	Pseudogene	n/a	-	-
CLI_2767	Hypothetical protein	-	-	-
CLI_2768	Pseudogene	n/a	-	-
CLI_2769	**LegC**	NPD4_3127	60	99
CLI_2770	**LegB**	NPD4_3129	78	98
CLI_2771	Acyltransferase	NPD4_3125	47	86
CLI_2772	*N*-acetylglucosamine deacetylase	-	-	-
CLI_2773	**LegF**	NPD4_3122	59	95
CLI_2774	CarB	-	-	-
CLI_2775	**LegI**	NPD4_3124	60	92
CLI_2776	Methyltransferase, PtmH ortholog	NPD4_3117	57	88
CLI_2777	**LegG**	NPD4_3123	61	99
CLI_2778	Nucleotidyl transferase	NPD4_3126	60	95
CLI_2779	Conserved hypothetical protein	NPD4_3106	63	90
CLI_2780	Conserved hypothetical protein	NPD4_3105	56	94
CLI_2781	Flagellin	NPD4_3130	75	100

[a]The FGI of *C. botulinum* strains contain two distinct regions, termed FGI-I and FGI-II (4). While FGI-I is mostly conserved across *C. botulinum*, the genetic content of FGI-II is highly divergent and responsible for the heterogeneity of flagellin glycans observed across strains (4). [b]Gene is not encoded within the conserved flagellar glycosylation cluster of *C. butyricum* CDC_51208.

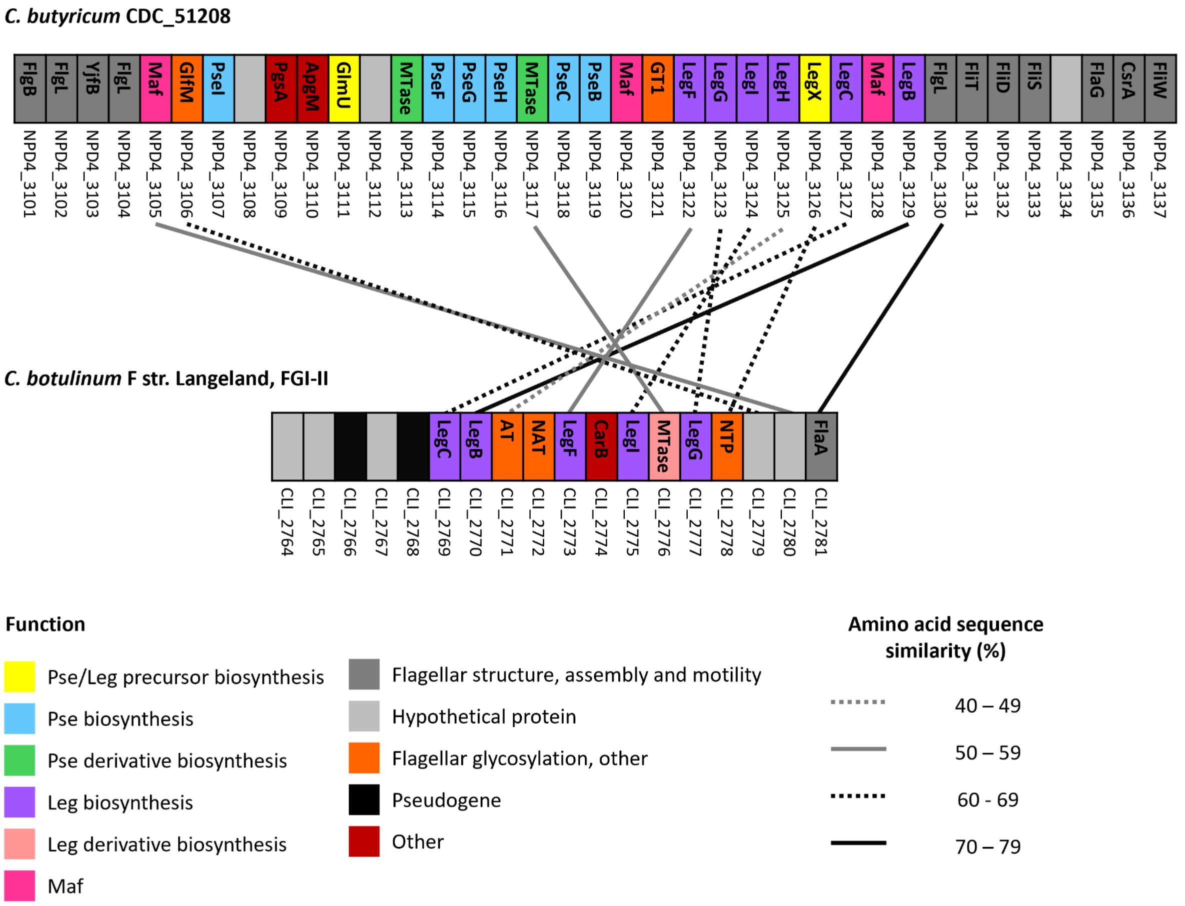

Figure S1. Genes encoded in the flagellar glycosylation locus of *C. butyricum* CDC_51208 and the FGI-II of *C. botulinum* F str. Langeland. *C. butyricum* CDC_51208 orthologues of genes present in the flagellar glycosylation locus of *C. botulinum* F str. Langeland are indicated with connecting lines that indicate the level of amino acid sequence similarity. AT, acetyltransferase; MTase, methyltransferase; NAT, *N*-acetyltransferase; NTP, nucleotidyltransferase.

Table S4. Amino acid sequence identity between each of the flagellin proteins encoded in the flagellar HVR of *C. butyricum* strain CDC_51208.

	**FlgL (NPD4_3102)**	**FlgL (NPD4_3104)**	**FlgL (NPD4_3130)**
	**Amino acid sequence identity (%)**		
**FlgL (NPD4_3102)**		30.6	34.6
**FlgL (NPD4_3104)**			49.6
**FlgL (NPD4_3130)**			

Table S5. Amino acid sequence identity between each of the Maf proteins encoded in the flagellar HVR of *C. butyricum* strain CDC_51208.

	Maf (NPD4_3105)	Maf (NPD4_3120)	Maf (NPD4_3128)
	Amino acid sequence identity (%)		
**Maf (NPD4_3105)**		28.7	26.6
**Maf (NPD4_3120)**			39.8
**Maf (NPD4_3128)**			

Table S6. Genes encoded within the flagellar HVR (flanked by *fliD* and *flgB*) of the 16 *C. butyricum* strains with complete genome assemblies. Strains are ordered by the number of genes present in the flagellar HVR, from smallest to largest. Genes highlighted in bold are predicted to be involved in the biosynthesis and transfer of the flagellin modification.

***C. butyricum* strain, GenBank Accession and Reference**	**Isolation source**	**Predicted Flagellin Modification**	**Genes in Flagellar HVR**	
			**Locus Tags**	**Predicted Function**
JKY6D1<br>GCA_001465175.1<br>(5)	Pit mud	None	-[a]	-
4-1<br>GCA_005145085.1<br>(6)	Human stool sample	None	FBD76_11380	FlgL, flagellin
			FBD76_11375	Yjfb family protein
			FBD76_11370	FlgL, flagellin
			FBD76_11365	FliT, FliD export chaperone
16-3<br>GCA_013112415.1<br>(7)	Human stool sample	None	HLV60_13315	Flagellin
			HLV60_13320	Yjfb family protein
			HLV60_13325	FlgL, flagellin
			HLV60_13330	FliT, FliD export chaperone
DKU-11<br>GCA_030389005.1<br>(8)	Human stool sample	None	QVH08_04520	FlgL, flagellin
			QVH08_04515	Yjfb family protein
			QVH08_04510	FlgL, flagellin
			QVH08_04505	FliT, FliD export chaperone
GBW-N1<br>GCA_041937605.1	Broiler tissue sample	None	ACEZ2L_03555	FlgL, flagellin
			ACEZ2L_03540	Yjfb family protein
			ACEZ2L_03545	FlgL, flagellin
			ACEZ2L_03540	FliT, FliD export chaperone
KNU-L09<br>GCA_001456065.2<br>(9)	Human stool sample	None	ATN24_04950	FlgL, flagellin
			ATN24_04945	Hypothetical protein
			ATN24_04940	FlgL, flagellin
			ATN24_04935	FliT, FliD export chaperone
LV1<br>GCA_027627495.1<br>(10)	Shrimp intestine	None	O4N14_03550	FlgL, flagellin
			O4N14_03545	Yjfb family protein
			O4N14_03540	FlgL, flagellin
			O4N14_03535	FliT, FliD export chaperone
TOA<br>GCA_001646605.1<br>(11)	Probiotics	None	AZ909_03385	FlgL, flagellin
			AZ909_03380	Hypothetical protein
			AZ909_03375	FlgL, flagellin
			AZ909_03370	FliT, FliD export chaperone
QXYZ514<br>GCA_026651935.1<br>(12)	Soil	None	C0637_RS02995	FlgL, flagellin
			C0637_RS02990	Yjfb family protein
			C0637_RS02985	FlgL, flagellin
			C0637_RS02980	FliT, FliD export chaperone
CBM588<br>GCA_030758275.1<br>(13)	Human stool sample	None	Q9978_04540	FlgL, flagellin
			Q9978_04535	Yjfb family protein
			Q9978_04530	Hypothetical protein
			Q9978_04525	FlgL, flagellin
			Q9978_04520	FliT, FliD export chaperone
CFSA3987<br>GCA_009650315.1<br>(14)	NEC case, stool sample	Fuc4N, Glc	EBL75_07700	FlgL, flagellin
			EBL75_07695	Yjfb family protein
			EBL75_07690	Methionyl-tRNA formyltransferase
			EBL75_07685	Lipid II:glycine glycyltransferase
			EBL75_07680	Hypothetical protein
			EBL75_07675	**RffA, dTDP-4-amino-4,6-dideoxygalactose transaminase**
			EBL75_07670	**RfbB, dTDP-glucose 4,6-dehydratase**
			EBL75_07665	**Radical SAM protein**
			EBL75_07660	**Flagellin glycosyltransferase**
			EBL75_07655	**Flagellin glycosyltransferase**
			EBL75_07650	FlgL, flagellin
			EBL75_07645	**Flagellin glycosyltransferase**

			EBL75_07640	FliT, FliD export chaperone
CFSA3989<br>GCA_009650335.1<br>(14)	NEC outbreak, environmental swab	Fuc4N, Glc	EBQ27_07705	FlgL, flagellin
			EBQ27_07700	Yjfb family protein
			EBQ27_07695	Methionyl-tRNA formyltransferase
			EBQ27_07690	Lipid II:glycine glycyltransferase
			EBQ27_07685	Hypothetical protein
			EBQ27_07680	**RffA, dTDP-4-amino-4,6-dideoxygalactose transaminase**
			EBQ27_07675	**RfbB, dTDP-glucose 4,6-dehydratase**
			EBQ27_07670	**Radical SAM protein**
			EBQ27_07665	**Flagellin glycosyltransferase**
			EBQ27_07660	**Flagellin glycosyltransferase**
			EBQ27_07655	**Flagellin glycosyltransferase**
			EBQ27_07650	FlgL, flagellin
			EBQ27_07645	**Flagellin glycosyltransferase**
			EBQ27_07640	FliT, FliD export chaperone
CBUT<br>GCA_018140655.1<br>(15)	Probiotics	Methylation	KDJ93_03730	FlgL, flagellin
			KDJ93_03725	YjfB family protein
			KDJ93_03720	PD-(D/E)XK nuclease domain-containing protein (pseudogene)
			KDJ93_03715	IS3 family transposase
			KDJ93_03710	IS982 family transposase
			KDJ93_03705	IS982 family transposase
			KDJ93_03700	ISL3 family transposase
			KDJ93_03695	Transposase
			KDJ93_03690	IS21-like element helper ATPase IstB
			KDJ93_03685	IS21 family transposase
			KDJ93_03680	Transposase
			KDJ93_03675	ISL3 family transposase
			KDJ93_03670	IS3 family transposase
			KDJ93_03665	IS256 family transposase
			KDJ93_03660	**FliB, flagellin lysine-*N*-methylase**
			KDJ93_03655	FlgL, flagellin
			KDJ93_03650	FliT, FliD export chaperone
DSM 10702<br>GCA_014131795.1<br>(16)	Pig intestine	Glc, Par	FF104_14340	IS256 family transposase
			FF104_14345	FliE, flagellar hook-basal body complex protein
			FF104_14350	FlgL, flagellin
			FF104_14355	**RfbH, CDP-4-dehydro-6-deoxyglucose reductase**
			FF104_14360	**RfbG, CDP-glucose 4,6-dehydratase**
			FF104_14365	**RfbS, CDP-paratose synthase**
			FF104_14370	3-dehydroquinate synthase
			FF104_14375	Beta-ketoacyl-ACP reductase
			FF104_14380	Citrate lyase family protein
			FF104_14385	Acetolactate synthase large subunit
			FF104_14390	**Class I SAM-dependent methyltransferase**
			FF104_14395	**RfbF, glucose-1-phosphate cytidylyltransferase**
			FF104_14400	**Flagellin glycosyltransferase**
			FF104_14405	**Flagellin glycosyltransferase**
			FF104_14410	**Flagellin glycosyltransferase**
			FF104_14415	FlgL, flagellin
			FF104_14420	YjfB motility family protein
			FF104_14425	FliT, FliD export chaperone
CFSA-TJ-E<br>GCA_024399875.1	Botulism case, stool sample	Fuc, Gal, Glc, Methylation	HNS01_03625	FlgL, flagellin

(17)			HNS01_03620	**Glycosyltransferase family 2 protein**
			HNS01_03615	**UDP-galactopyranose mutase**
			HNS01_03610	**GDP-fucose synthetase**
			HNS01_03605	**Radical SAM protein**
			HNS01_03600	**Glycosyltransferase family 2 protein**
			HNS01_03595	**dTDP-glucose 4,6-dehydratase**
			HNS01_03590	**HAD hydrolase-like protein**
			HNS01_03585	**Radical SAM protein**
			HNS01_03580	**GDP-fucose synthetase**
			HNS01_03575	**dTDP-glucose 4,6-dehydratase**
			HNS01_03570	**FkbM family methyltransferase**
			HNS01_03565	Transketolase
			HNS01_03560	Transketolase
			HNS01_03555	**RfbG, CDP-glucose 4,6-dehydratase**
			HNS01_03550	**RbfF, Glucose-1-phosphate cytidylyltransferase**
			HNS01_03545	**Flagellin glycosyltransferase**
			HNS01_03540	**FliB, flagellin lysine-*N*-methylase**
			HNS01_03535	FlgL, flagellin
CDC_51208 GCA_001886875.1 (2)	Botulism	Leg, Pse	NPD4_3102	FlgL, flagellin
			NPD4_3103	YjfB motility family protein
			NPD4_3104	FlgL, flagellin
			NPD4_3105	**Maf, motility-associated factor**
			NPD4_3106	**GlfM, Maf specificity factor**
			NPD4_3107	**PseI, Pseudaminic acid synthase**
			NPD4_3108	Hypothetical protein
			NPD4_3109	PgsA, CDP-alcohol phosphatidyltransferase
			NPD4_3110	ApgM, 2,3-bisphosphoglycerate-independent phosphoglycerate mutase
			NPD4_3111	**GlmU, *N*-acetylglucosamine-1-phosphate uridyltransferase**
			NPD4_3112	Hypothetical protein
			NPD4_3113	**SAM-dependent methyltransferase**
			NPD4_3114	**PseF, Pseudaminic acid cytidylyltransferase**
			NPD4_3115	**PseG, UDP-2,4-diacetamido-2,4, 6-trideoxy-beta-L-altropyranose hydrolase**
			NPD4_3116	**PseH, UDP-4-amino-4, 6-dideoxy-*N*-acetyl-beta-L-altrosa mine *N*-acetyltransferase**
			NPD4_3117	**Class I SAM-dependent methyltransferase**
			NPD4_3118	**PseC, UDP-4-amino-4,6-dideoxy-*N*-acetyl -beta-L-altrosamine transaminase**
			NPD4_3119	**PseB, UDP-*N*-acetylglucosamine 4,6-dehydratase**
			NPD4_3120	**Maf, motility-associated factor**
			NPD4_3121	**GT1 Glycosyltransferase**
			NPD4_3122	**LegF, CMP-*N,N′*-diacetyllegionaminic acid synthase**
			NPD4_3123	**LegG, GDP/UDP-*N,N′*-diacetylbacillosam ine 2-epimerase**
			NPD4_3124	**LegI, *N,N′*-diacetyllegionaminic acid synthase**

	NPD4_3125	**LegH, GDP-4-amino-4,6-dideoxy-α-D-*N*-acetylglucosamine *N*-acetyltransferase**
	NPD4_3126	**LegX, nucleotidyl transferase**
	NPD4_3127	**LegC, PLP-dependent aminotransferase**
	NPD4_3128	**Maf, motility-associated factor**
	NPD4_3129	**LegB, NAD-dependent GDP-*N*-acetylglucosamine 4,6-dehydratase**
	NPD4_3130	FlgL, flagellin
	NPD4_3131	FliT, FliD export chaperone

[a]A flagellar HVR is absent from the strain JKY6D1 due to a deletion in the flagellar biosynthesis locus.

Table S7. The GenBank accession numbers and isolation sources of the 98 *Clostridium* genomes included in the HVR content analysis, in addition to the locus tags of the genes encoding *flgB* and *fliD*, and an indication of the presence or absence of a flagellar HVR and an FGI in each genome. Reference genomes for each species are highlighted in bold and are presented as the first strain listed for each species.

Clostridium Species	Strain	GenBank Accession, Isolation Source and Reference	*flgB*	*fliD*	HVR	FGI
C. aceticum	**DSM 1496**	GCA_001042715.1 Sludge of town canal (18)	CACET_c20400[a]	CACET_c03000[a]	No	-
C. acetobutylicum	**DSM 1731**	GCA_000218855.1 DSMZ (19)	SMB_G2198	SMB_G2238	Yes	Yes
C. acetobutylicum	ATCC 824	GCA_000008765.1 Garden soil (20)	CA_C2165	CA_C2205	Yes	Yes
C. acetobutylicum	EA 2018	GCA_000191905.1 Soil, mutagenesis (21)	CEA_G2179	CEA_G2219	Yes	Yes
C. argentinense	**89G**	GCA_002074155.1 n/a (22)	RSJ17_12770	RSJ17_12895	Yes	Yes
C. autoethanogenum	**DSM 10061**	GCA_001484725.1 Rabbit faeces (23)	CLAU_3027	CLAU_2971	Yes	Yes
C. autoethanogenum	S232868	GCA_040166795.1 Rabbit faeces, lab adapted (24)	LAbrini_15550	LAbrini_15270	Yes	Yes
C. baratii	**CDC51267**	GCA_001991075.2 Infant botulism case (2)	Absent	Absent	No	-
C. baratii	C148	GCA_037066025.1 Red fox intestine	Absent	Absent	No	-
C. baratii	C162	GCA_037064645.1 Racoon intestine	Absent	Absent	No	-
C. baratii	C201	GCA_037059035.1 Red fox intestine	Absent	Absent	No	-
C. baratii	i39-0019-1A7	GCA_042851575.1 Human faeces (25)	Absent	Absent	No	-
C. beijerinckii	**DSM 791**	GCA_018223745.1 Fermentation bioreactor (26)	KEC93_21640	KEC93_21710	Yes	Yes
C. beijerinckii	BAS/B3/I/124	GCA_002003345.1 Industrial strain (27)	CLBIJ_45770	CLBIJ_45950	Yes	Yes
C. beijerinckii	CBEI	GCA_018140595.1 Probiotic formulation (15)	KDJ94_22380	KDJ94_22490	Yes	Yes
C. beijerinckii	CloBei18h	GCA_025962575.1 Cell pellet of fermenting cultures (28)	OD350_18535	OD350_18640	Yes	Yes
C. beijerinckii	NCIMB 8052	GCA_000016965.1 Soil (29)	Cbei_4271	Cbei_4291	Yes	Yes
C. bornimense	**M2/40**	GCA_000577895.1 Biogas reactor (30)	CM240_1648	CM240_1676	Yes	Yes
C. botulinum	**ATCC 3502**	GCA_000063585.1 Botulism case (31)	CBO2665	CBO2733	Yes	Yes
C. botulinum	ATCC 19397	GCA_000017025.1 Botulism case (32)	CLB_2608	CLB_2674	Yes	Yes
C. botulinum	E3 str. Alaska E43	GCA_000020285.1 Botulism case (33)	CLH_0804	CLH_0781	Yes	Yes
C. botulinum	F str. Langeland	GCA_000017065.1 Botulism case	CLI_2732	CLI_2783	Yes	Yes
C. cadaveris	**IFB3C5**	GCA_020911725.1 Adenocarcinoma in distal colon	KQH81_09505	KQH81_09550	Yes	Yes

		(34)				
C. carboxidivorans	**P7**	GCA_001038625.1<br>Agricultural settling lagoon<br>(35)	Ccar_25195	Ccar_25040	Yes	Yes
C. cellulovorans	**743B**	GCA_000145275.1<br>Cellulose digester<br>(36)	Clocel_1711	Clocel_1708	Yes	No
C. chauvoei	**12S0467**	GCA_002327205.1<br>Blackleg tissue sample, bovine<br>(37)	BTM20_05020	BTM20_04985	Yes	No
C. chauvoei	DSM 7528	GCA_002327185.1<br>Blackleg tissue sample, bovine<br>(37)	BTM21_08035	BTM21_08075	Yes	No
C. chauvoei	JF4335	GCA_900168365.1<br>Cattle with blackleg<br>(38)	CCH01_08710	CCH01_08640	Yes	No
C. chauvoei	SBP 07/09	GCA_004328885.1<br>Cattle muscle<br>(39)	C6H62_04260	C6H62_04225	Yes	No
C. cochlearium	**NCTC13027**	GCA_900187165.1<br>n/a	SAMEA4530647_01445	SAMEA4530647_01501	Yes	Yes
C. diolis	**DSM 15410**	GCA_008705175.1<br>Fermentation bioreactor<br>(40)	F3K33_21860	F3K33_21950	Yes	Yes
C. drakei	**SL1**	GCA_003096175.1<br>Environment<br>(41)	B9W14_04125	B9W14_03955	Yes	Yes
C. estertheticum	**CF001**	GCA_030585465.1<br>Bovine faeces<br>(42)	KTC99_17290	KTC99_17370	Yes	No
C. estertheticum	CEST001	GCA_027594565.1<br>Vacuum packed lamb<br>(43)	LOR37_17015	LOR37_17080	Yes	No
C. estertheticum	CF007	GCA_030585485.1<br>Bovine faeces<br>(42)	KTC98_19990	KTC98_20040	Yes	No
C. estertheticum	CM034	GCA_026650925.1<br>Horse meat<br>(44)	LL127_13140	LL127_13075	Yes	No
C. estertheticum	DSM 8809	GCA_001877035.1<br>Vacuum packed beef<br>(45)	A7L45_14255	A7L45_14370	Yes	Yes
C. felsineum	**DSM 794**	GCA_002006355.2<br>DSMZ<br>(27)	CLFE_020710	CLFE_020200	Yes	Yes
C. felsineum	DSM 6424	GCA_002006175.2<br>DSMZ<br>(27)	CLROS_016610	CLROS_016060	Yes	Yes
C. felsineum	DSM 793	GCA_002006235.2<br>DSMZ<br>(27)	CLAUR_009140	CLAUR_009650	Yes	Yes
C. fermenticellae	**JN500901**	GCA_003600355.1<br>Pit mud<br>(46)	D4Z93_09150	D4Z93_09280	Yes	Yes
C. formicaceticum	**DSM 92**	GCA_002080475.1<br>Sewers and ditches<br>(47)	CLFO_22690[a]	CLFO_03310[a]	No	-
C. formicaceticum	ATCC 27076	GCA_001854185.1<br>Sewage plant<br>(48)	BJL90_16595[a]	BJL90_07185[a]	No	-
C. gasigenes	**CGAS001**	GCA_017348895.1<br>Sheep meat<br>(49)	J1C67_00930	J1C67_00970	Yes	No
C. gelidum	**C5S11**	GCA_019977655.1<br>Rice field soil<br>(50)	psyc5s11_46660	psyc5s11_46960	Yes	Yes
C. intestinale	**PC17**	GCA_035905475.1<br>(51)	P8F83_11625	P8F83_11705	Yes	Yes
C. intestinale	Lx1	GCA_013781885.1<br>Cattle faeces<br>(52)	HZF06_16895	HZF06_16985	Yes	Yes
C. isatidis	**DSM 15098**	GCA_002285495.1<br>Woad vat	BEN51_06305	BEN51_06395	Yes	Yes
C. kluyveri	**NBRC 12016**	GCA_000010265.1<br>n/a	CKR_1048[a]	CKR_1852[a]	No	-

C. kluyveri	BJN0002	GCA_026240615.1<br>Cellar pit mud	OP486_01260[a]	OP486_21725[a]	No	-
C. kluyveri	DSM 555	GCA_000016505.1<br>Canal mud<br>(53)	CKL_1150[a]	CKL_2113[a]	No	-
C. kluyveri	JZZ	GCA_001902295.1<br>Pit mud<br>(54)	BS101_07175[a]	BS101_11365[a]	No	-
C. ljungdahlii	**DSM 13528**	GCA_000143685.1<br>Chicken yard waste<br>(55)	CLJU_c10190	CLJU_c09580	Yes	Yes
C. ljungdahlii	240531	GCA_045257525.1<br>n/a<br>(56)	ACI7YY_05425	ACI7YY_05095	Yes	Yes
C. ljungdahlii	delaor1	GCA_045257535.1<br>n/a<br>(56)	ACI7YX_05635	ACI7YX_05290	Yes	Yes
C. ljungdahlii	delaor2	GCA_045257545.1<br>n/a<br>(56)	ACI7YW_05170	ACI7YW_04870	Yes	Yes
C. ljungdahlii	delaor1aor2	GCA_045257575.1<br>n/a<br>(56)	ACI7YV_05175	ACI7YV_04855	Yes	Yes
C. manihotivorum	**CT4**	GCA_004015185.1<br>Cassava pulp and soil<br>(57)	C1I91_08435	C1I91_08315	Yes	Yes
C. novyi	**150557**	GCA_003614235.1<br>Swine liver<br>(58)	DFH04_09880	DFH04_09985	Yes	Yes
C. novyi	NT	GCA_000014125.1<br>Cancer therapeutic<br>(59)	NT01CX_1899	NT01CX_1879	Yes	Yes
C. pasteurianum	**ATCC 6013**	GCA_001856645.1<br>ATCC<br>(60,61)	AQ983_08795	AQ983_08605	Yes	Yes
C. pasteurianum	BC1	GCA_000389635.1<br>Coal-cleaning residues<br>(62)	Clopa_2914	Clopa_2957	Yes	Yes
C. pasteurianum	M150B	GCA_001856695.1<br>Mutated ATCC strain<br>(63)	AQ984_08785	AQ984_08595	Yes	Yes
C. pasteurianum	UCM B-7570	GCA_026410065.1<br>Fermenter media<br>(64)	OSC52_09645	OSC52_09370	Yes	Yes
C. perfringens	**FDAARGOS_903**	GCA_016027375.1<br>n/a<br>(65)	Absent	Absent	No	-
C. perfringens	ATCC 13124	GCA_000013285.1<br>Quality control organism for clinical tests<br>(66)	Absent	Absent	No	-
C. perfringens	EHE-NE18	GCA_003203455.1<br>Red junglefowl gut contents<br>(67)	Absent	Absent	No	-
C. perfringens	FORC_003	GCA_001304735.1<br>Aquarium water	Absent	Absent	No	-
C. perfringens	JP838	GCA_001579785.1<br>Dog with hemorrhagic gastroenteritis<br>(68)	Absent	Absent	No	-
C. saccharobutylicum	**DSM 13864**	GCA_000473995.1<br>Soy beans<br>(69)	CLSA_c39390	CLSA_c39650	Yes	Yes
C. saccharobutylicum	BAS/B3/SW/136	GCA_002003325.1<br>n/a<br>(27)	CSACC_39160	CSACC_39420	Yes	Yes
C. saccharobutylicum	NCP 195	GCA_002003385.1<br>n/a<br>(27)	CLOSACC_39160	CLOSACC_39420	Yes	Yes
C. saccharobutylicum	NCP 200	GCA_002003285.1<br>n/a<br>(27)	CLOSC_39110	CLOSC_39370	Yes	Yes
C. saccharobutylicum	NCP 258	GCA_002003365.1<br>n/a<br>(27)	CLOBY_38450	CLOBY_38710	Yes	Yes
C. saccharoper-butylacetonicum	**N1-4(HMT)**	GCA_000340885.1<br>Soil	Cspa_c45250	Cspa_c45730	Yes	Yes

		(70)				
C. saccharoper-butylacetonicum	N1-504	GCA_002003305.1<br>Soil<br>(27)	CLSAP_42870	CLSAP_43440	Yes	Yes
C. scatologenes	**ATCC 25775**	GCA_000968375.1<br>n/a<br>(71)	CSCA_3155	CSCA_3190	Yes	Yes
C. septicum	**DSM 7534**	GCA_003606265.1<br>DSMZ<br>(72)	CP523_02630	CP523_02670	Yes	No
C. septicum	RMA 8861	GCA_023988885.1<br>n/a	NH397_10965	NH397_11005	Yes	No
C. septicum	VAT12	GCA_004101825.1<br>Wild turkey<br>(72)	EI377_13165	EI377_13195	Yes	No
C. septicum	WW106	GCA_030664715.1<br>Wastewater<br>(73)	Q6375_11150	Q6375_11190	Yes	No
C. sporogenes	**NCIMB 10696**	GCA_000973705.1<br>Soil<br>(74)	CLSPOx_13725	CLSPOx_14045	Yes	Yes
C. sporogenes	AM1195	GCA_002865785.1<br>Infant botulism case<br>(75)	RSJ11_15405	RSJ11_15690	Yes	Yes
C. sporogenes	CDC_1632	GCA_001889325.1<br>Infant botulism case	NPD5_3271	NPD5_3211	Yes	Yes
C. sporogenes	CDC_67071	GCA_001886775.1<br>n/a	NPD7_417	NPD7_353	Yes	Yes
C. sporogenes	LHA6	GCA_021495975.1<br>As-contaminated paddy soil<br>(76)	L0894_13690	L0894_13990	Yes	Yes
C. taeniosporum	**1/k**	GCA_001735765.2<br>Lake silt<br>(77)	BGI42_03975	BGI42_03890	Yes	Yes
C. tagluense	**CM022**	GCA_026650945.1<br>Lamb<br>(44)	LL095_15105	LL095_15195	Yes	Yes
C. tagluense	CM008	GCA_030585445.1<br>Lamb meat<br>(44)	KTC93_16225	KTC93_16300	Yes	Yes
C. tetani	**E88**	GCA_000007625.1<br>Vaccine production strain<br>(78)	CTC_01678	CTC_01718	Yes	Yes
C. tetani	12124569	GCA_000967115.1<br>Human osteitis case<br>(79)	BN906_01814	BN906_01861	Yes	Yes
C. tetani	CMCC64008	GCA_029636225.1<br>Vaccine production strain for quality control	PAA20_08715	PAA20_08935	Yes	Yes
C. tetani	KHSU-134307-016	GCA_033127885.1<br>Soil<br>(80)	K134307016_16030	K134307016_16450	Yes	Yes
C. tetani	Mfbjulcb2	GCA_003013635.1<br>Fish market	C3B72_03395	C3B72_03605	Yes	Yes
C. thermo-succinogenes	**DSM 5807**	GCA_002896855.1<br>Beet pulp, sugar refinery<br>(81)	CDO33_07680[a]	CDO33_00725[a]	No	-
C. tyrobutyricum	**KCTC 5387**	GCA_001642655.1<br>n/a<br>(82)	CTK_C20350	CTK_C21410	Yes	Yes
C. tyrobutyricum	L319	GCA_014170115.1<br>Cow rumen<br>(83)	GTH52_02440	GTH52_01930	Yes	Yes
C. tyrobutyricum	W428	GCA_001679705.1<br>Cow rumen<br>(84)	BA182_09175	BA182_09670	Yes	Yes

[a]The genes which encode FlgB and FliD do not flank a HVR and are each instead located at distant loci in the genome.

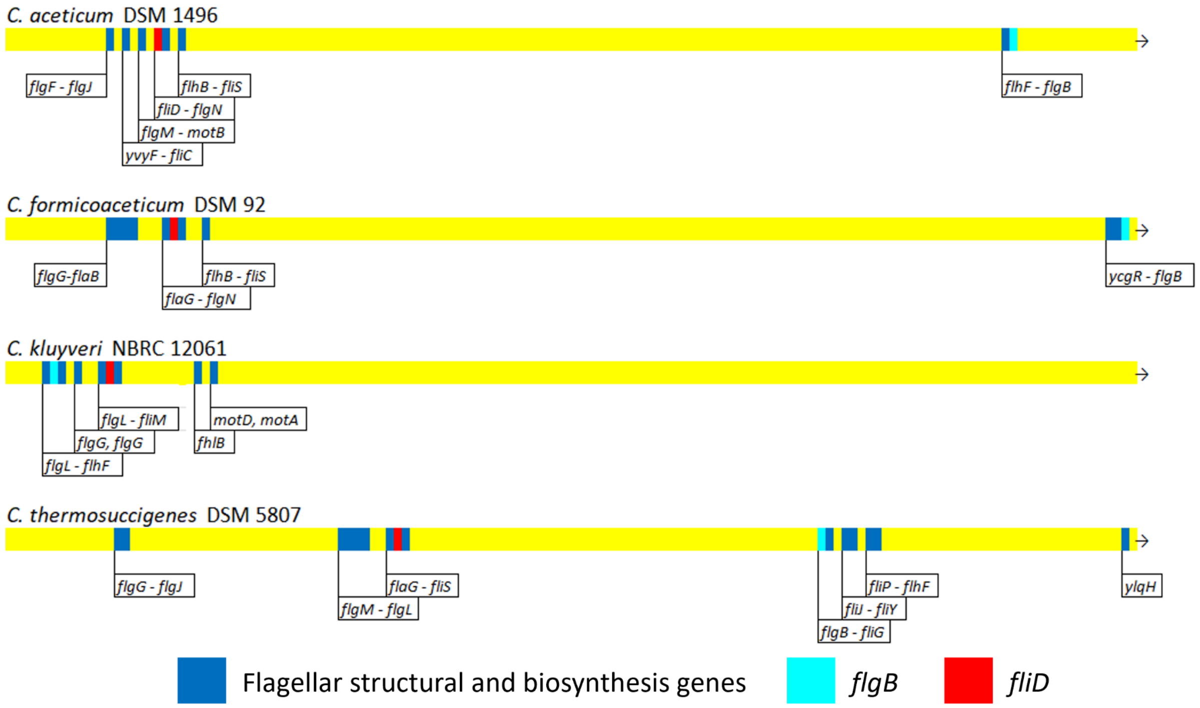

Figure S2. The distribution of flagellar structural and biosynthetic genes across the genomes of the species *C. aceticum*, *C. formicoaceticum*, *C. kluyveri* and *C. thermosuccinogenes*. The genes which encode FlgB and FliD, the genes which flank the flagellar HVR in the other *Clostridium* species included in our analysis, are shown in turquoise and red, respectively.

Table S8. The niche of each of the *Clostridium* species 28 reference genomes that contain an FGI, in addition to the nature of the NulO that is predicted to be encoded by the genes in this region. The 'niche' field was populated using the isolation source data recorded in Table S7.

***Clostridium* Species Reference Strains**	**Niche**	**Predicted NulO Flagellar Modification**
C. acetobutylicum DSM 1731	Environment/industry	Pse
C. argentinense 89G	Host associated, pathogen	Pse
C. autoethanogenum DSM 10061	Host associated, commensal	-
C. beijerinckii DSM 791	Environment/industry	Pse
C. bornimense M2/40	Environment/industry	Pse
C. botulinum A str. ATCC 3502	Host associated, pathogen	Pse
C. cadaveris IFB3C5	Host associated, pathogen	-
C. carboxidivorans P7	Environment/industry	Pse
C. cochlearium NCTC13027	Environment/industry	Pse
C. diolis DSM 15410	Environment/industry	PseAm
C. drakei SL1	Environment/industry	PseAm
C. felsineum DSM 794	Environment/industry	Pse
C. fermenticellae JN500901	Environment/industry	Pse
C. gelidum C5S11	Environment/industry	-
C. intestinale PC17	Host associated, commensal	-
C. isatidis DSM 15098	Environment/industry	-
C. ljungdahlii DSM 13528	Host associated, commensal	-
C. manihotivorum CT4	Environment/industry	Leg
C. novyi 150557	Host associated, commensal	PseAm
C. pasteurianum ATCC 6013	Environment/industry	-
C. saccharobutylicum DSM 13864	Environment/industry	PseAm
C. saccharoperbutylacetonicum N1-4(HMT)	Environment/industry	Pse
C. scatologenes ATCC 25775	Environment/industry	Pse
C. sporogenes NCUMB 10696	Environment/industry	Pse
C. taeniosporum 1/k	Environment/industry	Pse
C. tagluense CM022	Host associated, commensal	Pse
C. tetani E88	Host associated, pathogen	Pse
C. tyrobutyricum KCTC 5387	Environment/industry	-

Table S9. CMP-Pse biosynthetic genes encoded outside the flagellar HVR in the genomes of *C. autothanogenum* DSM 10061 and *C. ljungdahlii* DSM 13528, in addition to the locus tags of transposase sequences that are encoded in close proximity to these genes.

	***C. autoethanogenum* DSM 10061**	***C. ljungdahlii* DSM 13528**
PseB	CLAU_2559	CLJU_c05480
PseC	CLAU_2560	CLJU_c05490
PseH	CLAU_2561	CLJU_c05500
PseG	CLAU_2563	CLJU_c05520
PseI	CLAU_2562	CLJU_c05510
PseF	CLAU_2558	CLJU_c05470
Neighbouring transposase sequences	CLAU_2551	CLJU_c05380

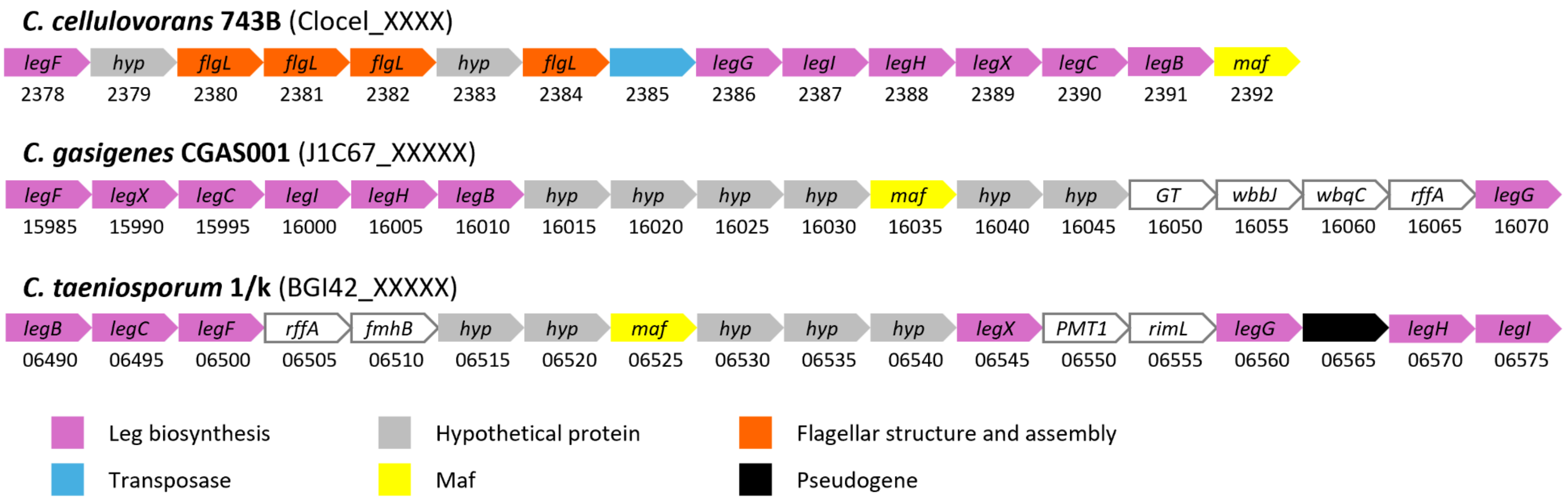

Figure S3. The organisation of CMP-Leg biosynthetic genes, which are predicted to be involved in flagellar glycosylation, but which are located outside the flagellar HVR in the genomes of the strains *C. cellulovorans* 743B, *C. gasigenes* CGAS001 and *C. taeniosporum* 1/k

Table S10. The locus tags and products of genes that are predicted to be involved in flagellar glycosylation in the species *C. formicaceticum*, *C. kluyveri* and *C. thermosuccigenes*, which do not possess flagellar HVRs.

***Clostridium* species**	**Strain**	**Locus Tag**	**Predicted Product**	**Predicted Flagellin Modification**
C. formicoaceticum	DSM 92	CLFO_02750	Flagellin B	Leg, Pse
		CLFO_02760	Hypothetical protein	
		CLFO_02770	Maf	
		CLFO_02780	LegB	
		CLFO_02790	LegC	
		CLFO_02800	LegH	
		CLFO_02810	AcpP	
		CLFO_02820	3-oxoacyl-[acyl-carrier-protein] synthase 3	
		CLFO_02830	3-oxoacyl-[acyl-carrier-protein] reductase FabG	
		CLFO_02840	Hypothetical protein	
		CLFO_02850	3-oxoacyl-[acyl-carrier-protein] reductase FabG	
		CLFO_02860	Long-chain-fatty-acid--CoA ligase	
		CLFO_02870	Holo-[acyl-carrier-protein] synthase	
		CLFO_02880	LegG	
		CLFO_02890	LegI	
		CLFO_02900	LegF	
		CLFO_02910	Glycosyltransferase	
		CLFO_02920	RfbB	
		CLFO_02930	Aminotransferase	
		CLFO_02940	Hypothetical protein	
		CLFO_02950	PseB	
		CLFO_02960	PseF	
		CLFO_02970	Molybdenum cofactor biosynthesis protein A	
		CLFO_02980	PseH	
		CLFO_02990	PseI	
		CLFO_03000	WbqC	
		CLFO_03010	LmbE	
		CLFO_03020	PseG	
		CLFO_03030	PseC	
		CLFO_03040	Radical SAM protein	
		CLFO_03050	Hypothetical protein	
		CLFO_03060	Hypothetical protein	
		CLFO_03070	Hypothetical protein	
		CLFO_03080	Hypothetical protein	
		CLFO_03090	Hypothetical protein	
		CLFO_03100	Chemotaxis protein PomA	
		CLFO_03110	Motility protein B	
		CLFO_32140	Maf	
		CLFO_32150	Hypothetical protein	
		CLFO_32160	Hypothetical protein	
		CLFO_32170	LegB	
		CLFO_32180	LegF	
		CLFO_32190	Gfo/Idh/MocA family oxidoreductase	
		CLFO_32200	LegX	
		CLFO_32210	LegH	
		CLFO_32220	LegI	
		CLFO_32230	LegG	
		CLFO_32240	LegC	
C. kluyveri	NBRC 12016	CKR_1832	Transposase	Leg
		CKR_1833	Hypothetical protein	
		CKR_1834	Maf	
		CKR_1835	Hypothetical protein	
		CKR_1836	PseB	

		CKR_1837	GDP-mannose 4,6-dehydratase	
		CKR_1838	UDP-glucose 6-dehydrogenase	
		CKR_1839	Alpha-amylase	
		CKR_1840	Biotin decarboxylase	
		CKR_1841	3-oxoacyl-ACP reductase	
		CKR_1842	Acetolactate synthase large subunit, IlvB	
		CKR_1843	Radical SAM domain protein	
		CKR_1844	Methyltransferase	
		CKR_1845	Bifunctional glycosyltransferase/methyltransferase	
		CKR_1846	Methyltransferase	
		CKR_1847	PseG	
		CKR_1848	PseI	
		CKR_1849	Maf	
		CKR_1850	Flagellin	
		CKR_1851	FliT	
		CKR_1852	FliD	
		CKR_2180	LegF	
		CKR_2181	LegX	
		CKR_2182	LegC	
		CKR_2183	LmbE	
		CKR_2184	LegG	
		CKR_2185	Acetyltransferase	
		CKR_2186	LegI	
		CKR_2187	LegH	
		CKR_2188	LegB	
		CKR_2189	Hypothetical protein	
		CKR_2190	Hypothetical protein	
		CKR_2191	Maf	
C. thermosuccigenes	DSM 5807	CDO33_00645	Flagellin	Glucose, a 3,6-dideoxy-hexose sugar
		CDO33_00655	CsrA	
		CDO33_00660	Flagellin	
		CDO33_00665	Flagellin glycosyltransferase	
		CDO33_00670	Glycosyltransferase	
		CDO33_00675	RfbF	
		CDO33_00680	RfbG	
		CDO33_00685	RfbH	
		CDO33_00690	RfbJ/RfbS	
		CDO33_00695	Methyltransferase	
		CDO33_00700	Transketolase	
		CDO33_00705	Transketolase	
		CDO33_00710	RfbB	
		CDO33_00715	RfbA	
		CDO33_00720	FlaG	
		CDO33_00725	FliD	

Table S11. The GenBank accessions and isolation sources of *C. butyricum* strains that have incomplete genome assemblies and encode complete CMP-Leg biosynthetic pathways in their FGIs.

***C. butyricum* Strain, GenBank Accession and Reference**	**Isolation Source**	**Locus Tag**	**Predicted Product**
YIM B08182<br>GCA_040813805.1	*Paris polyphylla* var. yunnanensis root	AB2T71_03845	FlgB
		AB2T71_03840	Flagellin
		AB2T71_03835	NAD-dependent epimerase/dehydratase
		AB2T71_03830	Thiamine pyrophosphate-binding protein
		AB2T71_03825	RfbH
		AB2T71_03820	RfbG
		AB2T71_03815	RfbF
		AB2T71_03810	Glycosyltransferase
		AB2T71_03805	**LegF**
		AB2T71_03800	**LegG**
		AB2T71_03795	**LegI**
		AB2T71_03790	**LegH**
		AB2T71_03785	**LegX**
		AB2T71_03780	**LegC**
		AB2T71_03775	**LegB**
		AB2T71_03770	Hypothetical protein
		AB2T71_03765	**Maf**
		AB2T71_03760	Flagellin
		AB2T71_03755	FliT
		AB2T71_03750	Hypothetical protein
		AB2T71_03745	FliD
L3_063_040G1_dasL3_063_040G1<br>GCA_018372835.1<br>(85)	Preterm infant, fecal sample	KIC67_08405	FlgB
		KIC67_08410	Flagellin
		KIC67_08415	**LegF**
		KIC67_08420	**LegG**
		KIC67_08425	**LegI**
		KIC67_08430	**LegH**
		KIC67_08435	**LegX**
		KIC67_08440	Radical SAM protein
		KIC67_08445	**LegC**
		KIC67_08450	**LegB**
		KIC67_08455	Hypothetical protein
		KIC67_08460	**Maf**
		KIC67_08465	Flagellin
		KIC67_08470	YjfB
		KIC67_08475	Hypothetical protein
		KIC67_08480	FliD
C420<br>GCA_050585425.1	Giant panda, fecal sample	ACQPUI_16305	FlgB
		ACQPUI_16300	Flagellin
		ACQPUI_16295	YjfB
		ACQPUI_16290	**RfbB**
		ACQPUI_16285	**RfbH**
		ACQPUI_16280	**RfbG**
		ACQPUI_16275	**RfbF**
		ACQPUI_16270	Glycosyltransferase
		ACQPUI_16265	**LegC**
		ACQPUI_16260	**LegB**
		ACQPUI_16255	**LegF**

	ACQPUI_16250	**LegG**
	ACQPUI_16245	**LegI**
	ACQPUI_16240	**LegH**
	ACQPUI_16235	**LegX**
	ACQPUI_16230	Radical SAM protein
	ACQPUI_16225	Hypothetical protein
	ACQPUI_16220	**Maf**
	ACQPUI_16215	Flagellin
	ACQPUI_16210	Hypothetical protein
	ACQPUI_16205	FliD

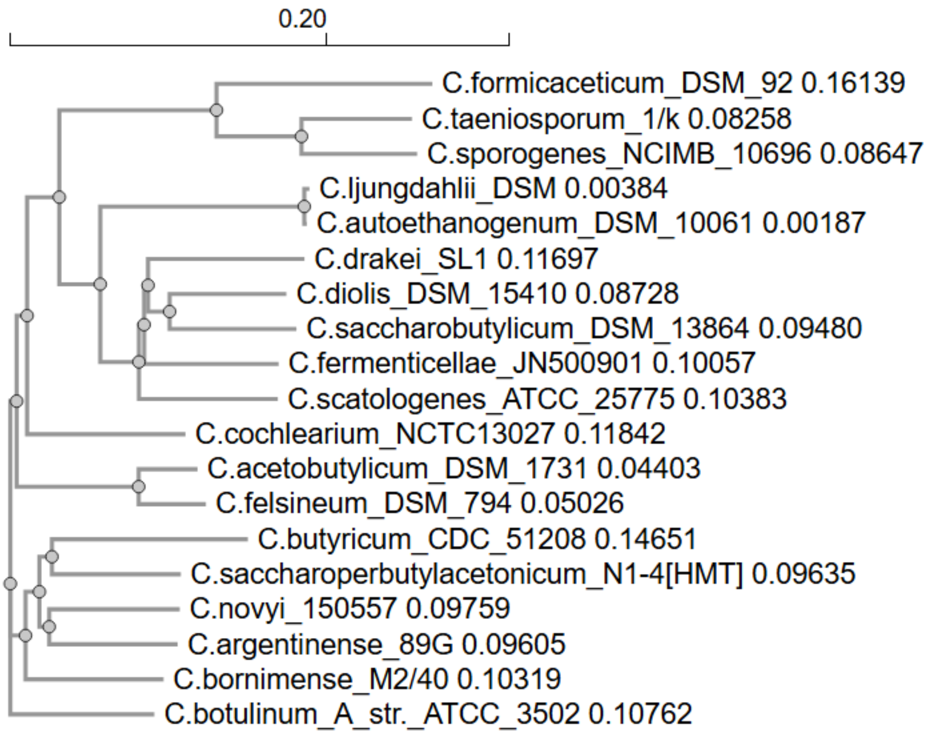

Figure S4. A phylogram based on the sequence alignments of PseI proteins of *Clostridium* species. Branch lengths indicate the extent of genetic change.

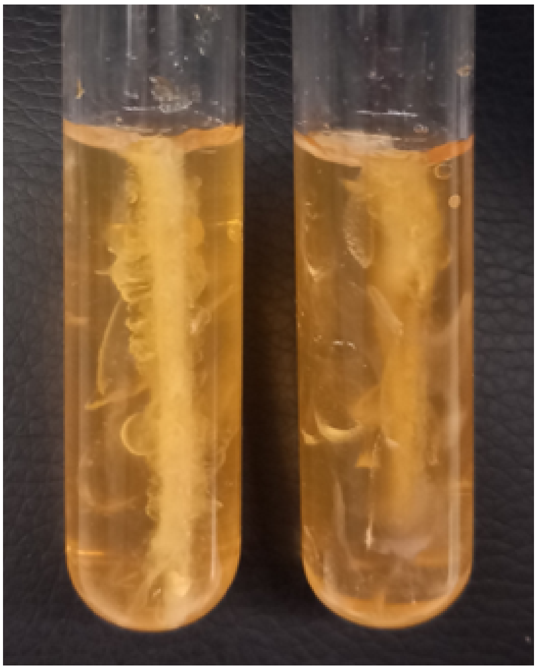

Figure S5. Motility stabs of a known motile strain of *C. acetobutylicum,* namely ATCC 824 (left) and *C. butyricum* DSM 10702 (right). Motility is visible by the movement of bacteria away from the vertical puncture axis, which resulted in an area of turbidity after 48 hours of incubation.

References

1. Hassan KA, Elbourne LDH, Tetu SG, Johnson EA, Paulsen IT. Genome sequence of the neurotoxigenic *Clostridium butyricum* strain 5521. Genome Announc [Internet]. 2014;2(3). Available from: http://dx.doi.org/10.1128/genomeA.00632-14

2. Halpin JL, Hill K, Johnson SL, Bruce DC, Brian Shirey T, Dykes JK, et al. Finished Whole-Genome Sequences of *Clostridium butyricum* Toxin Subtype E4 and *Clostridium baratii* Toxin Subtype F7 Strains. Genome Announc [Internet]. 2017 Jul 1;5(29). Available from: https://pubmed.ncbi.nlm.nih.gov/28729254/

3. Kwok JSL, Ip M, Chan TF, Lam WY, Tsui SKW. Draft genome sequence of *Clostridium butyricum* strain NOR 33234, isolated from an elderly patient with diarrhea. Genome Announc [Internet]. 2014 Dec 24 [cited 2025 Apr 2];2(6). Available from: https://pubmed.ncbi.nlm.nih.gov/25540356/

4. Carter AT, Paul CJ, Mason DR, Twine SM, Alston MJ, Logan SM, et al. Independent evolution of neurotoxin and flagellar genetic loci in proteolytic *Clostridium botulinum*. BMC Genomics. 2009 Mar 19;10(1):1–18.

5. Li C, Wang Y, Xie G, Peng B, Zhang B, Chen W, et al. Complete genome sequence of *Clostridium butyricum* JKY6D1 isolated from the pit mud of a Chinese flavor liquor-making factory. J Biotechnol. 2016 Feb 20;220:23–4.

6. Bang MS, Jeong HW, Lee YJ, Lee SC, Lee GS, Kim S, et al. Complete Genome Sequence of *Clostridium butyricum* Strain DKU_butyricum 4-1, Isolated from Infant Feces. Microbiol Resour Announc [Internet]. 2020 Mar 5;9(10). Available from: https://www.ncbi.nlm.nih.gov/pmc/articles/PMC7171215/

7. Shin JI, Bang MS, Lee GS, Kim HN, Oh CH. Draft Genome Sequence of *Clostridium butyricum* Strain 16-3, Isolated from Neonatal Feces. Microbiol Resour Announc [Internet]. 2022 Jul 18;11(8). Available from: http://dx.doi.org/10.1128/MRA.00163-22

8. Mo S. Complete genome sequence of *Clostridium butyricum* DKU-11, isolated from healthy infant feces. Microbiol Resour Announc. 2024 Jun 11;13(6):e0003724.

9. Shin J, Song Y, Jeong Y, Cho BK. Analysis of the core genome and pan-genome of autotrophic acetogenic bacteria [Internet]. Vol. 7, Frontiers in Microbiology. Frontiers Media S.A.; 2016. Available from: http://dx.doi.org/10.3389/fmicb.2016.01531

10. Wang Q, Li W, Liu H, Tan B, Dong X, Chi S, et al. The isolation, identification, whole-genome sequencing of *Clostridium butyricum* LV1 and its effects on growth performance, immune response, and disease-resistance of Litopenaeus vannamei. Microbiol Res. 2023 Jul 1;272:127384.

11. Isono A, Katsuno T, Sato T, Nakagawa T, Kato Y, Sato N, et al. *Clostridium butyricum* TO-A culture supernatant downregulates TLR4 in human colonic epithelial cells. Digestive Diseases and Sciences. 2007 Nov 3;52(11):2963–71.

12. Yang M, Zabed HM, Yun J, Zhang G, Qi ·X. The Draft Genome Sequence of *Clostridium*

butyricum QXYZ514, a Potent Bacterium for Converting Glycerol into Fuels and Bioproducts in the Waste-Based Biorefinery. Curr Microbiol. 2020;77:3371–6.

13. Wood L, Omorotionmwan BB, Blanchard AM, Dowle A, Bishop AL, Griffin R. Characterisation of the butyrate production pathway in probiotic MIYAIRI588 by a combined whole genome-proteome approach. bioRxivorg. 2023 Aug 20;2023.08.20.554021.

14. Dong Y, Li Y, Zhang DBA, Nguyen S, Maheshwari N, Hu Y, et al. Epidemiological and genetic characterization of *Clostridium butyricum* cultured from neonatal cases of necrotizing enterocolitis in China. 2020; Available from: https://doi.org/10.1017/ice.2019.289

15. Perraudeau F, McMurdie P, Bullard J, Cheng A, Cutcliffe C, Deo A, et al. Improvements to postprandial glucose control in subjects with type 2 diabetes: a multicenter, double blind, randomized placebo-controlled trial of a novel probiotic formulation. BMJ Open Diabetes Res Care. 2020 Jul 1;8(1):e001319.

16. Xin B, Tao F, Wang Y, Gao C, Ma C, Xu P. Genome Sequence of *Clostridium butyricum* Strain DSM 10702, a Promising Producer of Biofuels and Biochemicals. Genome Announc. 2013;1(4):563–76.

17. Dong Y, Wang W, Jiang T, Xu J, Li F. Whole genome sequencing of *Clostridium butyricum* that caused the first infant botulism in China. Disease Surveillance. 2022;(1):38–44.

18. Poehlein A, Bengelsdorf FR, Schiel-Bengelsdorf B, Gottschalk G, Daniel R, Dürre P. Complete genome sequence of Rnf- and cytochrome-containing autotrophic acetogen *Clostridium aceticum* DSM 1496. Genome Announc. 2015 Aug 27;3(4):e00786–15.

19. Bao G, Wang R, Zhu Y, Dong H, Mao S, Zhang Y, et al. Complete genome sequence of *Clostridium acetobutylicum* DSM 1731, a solvent-producing strain with multireplicon genome architecture. J Bacteriol. 2011 Sep;193(18):5007–8.

20. Nölling J, Breton G, Omelchenko MV, Makarova KS, Zeng Q, Gibson G, et al. Genome sequence and comparative analysis of the solvent-producing bacterium *Clostridium acetobutylicum*. Journal of Bacteriology. 2001;183(16):4823–38.

21. Hu S, Zheng H, Gu Y, Zhao J, Zhang W, Yang Y, et al. Comparative genomic and transcriptomic analysis revealed genetic characteristics related to solvent formation and xylose utilization in *Clostridium acetobutylicum* EA 2018. BMC Genomics. 2011 Feb 2;12(1):93.

22. Halpin JL, Hill K, Johnson SL, Bruce DC, Shirey TB, Dykes JK, et al. Finished whole-genome sequence of *Clostridium argentinense* producing botulinum neurotoxin type G. Genome Announc [Internet]. 2017 May 25;5(21). Available from: http://dx.doi.org/10.1128/genomeA.00380-17

23. Humphreys CM, McLean S, Schatschneider S, Millat T, Henstra AM, Annan FJ, et al. Whole genome sequence and manual annotation of *Clostridium autoethanogenum*, an industrially relevant bacterium. BMC Genomics. 2015 Dec 21;16(1):1085.

24. Ingelman H, Heffernan JK, Harris A, Brown SD, Shaikh KM, Saqib AY, et al. Autotrophic adaptive laboratory evolution of the acetogen *Clostridium autoethanogenum* delivers the

gas-fermenting strain LAbrini with superior growth, products, and robustness. N Biotechnol. 2024 Nov 25;83:1–15.

25. Honda K, Furuichi M, Kawaguchi T, Pust MM, Yasuma-Mitobe K, Plichta D, et al. Rationally-defined microbial consortia suppress multidrug-resistant proinflammatory Enterobacteriaceae via ecological control. Res Sq [Internet]. 2023 Oct 23 [cited 2025 Jun 17]; Available from: http://dx.doi.org/10.21203/rs.3.rs-3462622/v1

26. Sedlar K, Nykrynova M, Bezdicek M, Branska B, Lengerova M, Patakova P, et al. Diversity and evolution of *Clostridium beijerinckii* and complete genome of the type strain DSM 791T. Processes (Basel). 2021 Jul 10;9(7):1196.

27. Poehlein A, Solano JDM, Flitsch SK, Krabben P, Winzer K, Reid SJ, et al. Microbial solvent formation revisited by comparative genome analysis. Biotechnol Biofuels. 2017 Mar 9;10(1):58.

28. Zhang C, Li T, He J. Characterization and genome analysis of a butanol-isopropanol-producing *Clostridium beijerinckii* strain BGS1. Biotechnol Biofuels. 2018 Oct 11;11(1):280.

29. Wang Y, Li X, Mao Y, Blaschek HP. Single-nucleotide resolution analysis of the transcriptome structure of *Clostridium beijerinckii* NCIMB 8052 using RNA-Seq. BMC Genomics. 2011 Sep 30;12(1):479.

30. Hahnke S, Wibberg D, Tomazetto G, Pühler A, Klocke M, Schlüterb A. Whole genome sequence of *Clostridium bornimense* strain M2/40 isolated from a lab-scale mesophilic two-phase biogas reactor digesting maize silage and wheat straw. J Biotechnol. 2014 Aug 20;184:199–200.

31. Sebaihia M, Peck MW, Minton NP, Thomson NR, Holden MTG, Mitchell WJ, et al. Genome sequence of a proteolytic (Group I) *Clostridium botulinum* strain Hall A and comparative analysis of the clostridial genomes. Genome Res. 2007 Jul;17(7):1082–92.

32. Smith TJ, Hill KK, Foley BT, Detter JC, Munk AC, Bruce DC, et al. Analysis of the neurotoxin complex genes in *Clostridium botulinum* A1-A4 and B1 strains: BoNT/A3, /Ba4 and /B1 clusters are located within plasmids. PLoS One. 2007 Dec 5;2(12):e1271.

33. Söderholm H, Jaakkola K, Somervuo P, Laine P, Auvinen P, Paulin L, et al. Comparison of *Clostridium botulinum* genomes shows the absence of cold shock protein coding genes in type E neurotoxin producing strains. Botulinum J. 2013;2(3/4):189.

34. McGlinchey AS, Zepeda-Rivera MA, Stepanovica M, Baryiames AA, Jones DS, LaCourse KD, et al. Complete genome sequence of *Clostridium cadaveris* IFB3C5, isolated from a human colonic adenocarcinoma. Microbiol Resour Announc. 2022 Mar 17;11(3):e0113521.

35. Li N, Yang J, Chai C, Yang S, Jiang W, Gu Y. Complete genome sequence of *Clostridium carboxidivorans* P7(T), a syngas-fermenting bacterium capable of producing long-chain alcohols. J Biotechnol. 2015 Oct 10;211:44–5.

36. Tamaru Y, Miyake H, Kuroda K, Nakanishi A, Kawade Y, Yamamoto K, et al. Genome sequence of the cellulosome-producing mesophilic organism *Clostridium cellulovorans* 743B. J Bacteriol. 2010 Feb;192(3):901–2.

37. Thomas P, Semmler T, Eichhorn I, Lübke-Becker A, Werckenthin C, Abdel-Glil MY, et al. First report of two complete *Clostridium chauvoei* genome sequences and detailed in silico genome analysis. Infect Genet Evol. 2017 Oct 1;54:287–98.

38. Falquet L, Calderon-Copete SP, Frey J. Draft genome sequence of the virulent *Clostridium chauvoei* reference strain JF4335. Genome Announc [Internet]. 2013 Aug 15;1(4). Available from: http://dx.doi.org/10.1128/genomea.00593-13

39. Ziech RE, Farias LD, Balzan C, Luz MAB da, Frey J, Vargas AC de. Protective efficacy of commercial vaccines against a virulent field strain of *Clostridium chauvoei*. Semin Cienc Agrar. 2019 Jul 4;40(5):1837.

40. Sedlar K, Vasylkivska M, Musilova J, Branska B, Provaznik I, Patakova P. Phenotypic and genomic analysis of isopropanol and 1,3-propanediol producer *Clostridium diolis* DSM 15410. Genomics. 2021 Jan;113(1 Pt 2):1109–19.

41. Jeong Y, Song Y, Shin HS, Cho BK. Draft genome sequence of acid-tolerant *Clostridium drakei* SL1T, a potential chemical producer through syngas fermentation. Genome Announc [Internet]. 2014 May 15;2(3). Available from: http://dx.doi.org/10.1128/genomeA.00387-14

42. Wambui J, Cernela N, Stevens MJA, Stephan R. Whole genome sequence-based identification of *Clostridium estertheticum* complex strains supports the need for taxonomic reclassification within the species *Clostridium estertheticum*. Front Microbiol. 2021 Sep 13;12:727022.

43. Draft Genome Sequence of *Clostridium estertheticum* CEST001, Belonging to a Novel Subspecies of *C. estertheticum*, Isolated from Chilled Vacuum-Packed Lamb Meat Imported to Switzerland. Microbiology Resource Announcements [Internet]. 2020 Aug 13 [cited 2025 Jun 18];9(33). Available from: http://dx.doi.org/10.1128/mra.00806-20

44. Wambui J, Stevens MJA, Sieber S, Cernela N, Perreten V, Stephan R. Targeted genome mining reveals the psychrophilic *Clostridium estertheticum* complex as a potential source for novel bacteriocins, including cesin A and estercticin A. Front Microbiol. 2021;12:801467.

45. Yu Z, Gunn L, Brennan E, Reid R, Wall PG, Gaora PÓ, et al. Complete genome sequence of *Clostridium estertheticum* DSM 8809, a microbe identified in spoiled vacuum packed beef. Front Microbiol. 2016 Nov 11;7:1764.

46. Xu PX, Chai LJ, Qiu T, Zhang XJ, Lu ZM, Xiao C, et al. *Clostridium fermenticellae* sp. nov., isolated from the mud in a fermentation cellar for the production of the Chinese liquor, baijiu. Int J Syst Evol Microbiol. 2019 Mar;69(3):859–65.

47. Karl MM, Poehlein A, Bengelsdorf FR, Daniel R, Dürre P. Complete genome sequence of the autotrophic acetogen *Clostridium formicaceticum* DSM 92T using nanopore and illumina sequencing data. Genome Announc [Internet]. 2017 May 25;5(21). Available from: http://dx.doi.org/10.1128/genomea.00423-17

48. Bao T, Cheng C, Xin X, Wang J, Wang M, Yang ST. Deciphering mixotrophic *Clostridium formicoaceticum* metabolism and energy conservation: Genomic analysis and experimental studies. Genomics. 2019 Dec;111(6):1687–94.

49. Wambui J, Cernela N, Corti S, Stephan R. Comparative genome analysis and phenotypic characterization of *Clostridium gasigenes* CGAS001 isolated from chilled vacuum-packed lamb meat. Front Microbiol. 2020 Aug 24;11:2048.

50. Honma S, Ueki A, Tonouchi A, Kaku N, Ueki K. *Clostridium gelidum* sp. nov., a psychrotrophic anaerobic bacterium isolated from rice field soil. Int J Syst Evol Microbiol [Internet]. 2022 Aug [cited 2025 Jun 18];72(8). Available from: http://dx.doi.org/10.1099/ijsem.0.005478

51. Lubin JB, Green J, Maddux S, Denu L, Duranova T, Lanza M, et al. Arresting microbiome development limits immune system maturation and resistance to infection in mice. Cell Host Microbe. 2023 Apr 12;31(4):554–70.e7.

52. Liu X, Ye Y, Yang N, Cheng C, Rensing C, Jin C, et al. Nonelectroactive *Clostridium* obtains extracellular electron transfer-capability after forming chimera with *Geobacter*. ISME Commun. 2024 Jan;4(1):ycae058.

53. Seedorf H, Fricke WF, Veith B, Brüggemann H, Liesegang H, Strittmatter A, et al. The genome of *Clostridium kluyveri*, a strict anaerobe with unique metabolic features. Proc Natl Acad Sci U S A. 2008 Feb 12;105(6):2128–33.

54. Wang Y, Li B, Dong H, Huang X, Chen R, Chen X, et al. Complete genome sequence of *Clostridium kluyveri* JZZ applied in Chinese strong-flavor liquor production. Curr Microbiol. 2018 Jul 20;75(11):1429–33.

55. Köpke M, Held C, Hujer S, Liesegang H, Wiezer A, Wollherr A, et al. *Clostridium ljungdahlii* represents a microbial production platform based on syngas. Proc Natl Acad Sci U S A. 2010 Jul 20;107(29):13087–92.

56. Baur ST, Schulz S, McCluskey JB, Velázquez Gómez JA, Angenent LT, Molitor B. Deletion of aldehyde:ferredoxin oxidoreductase-encoding genes in *Clostridium ljungdahlii* results in changes in product spectrum with various carbon sources. Bioresour Technol. 2025 Sep;431(132596):132596.

57. Cheawchanlertfa P, Sutheeworapong S, Jenjaroenpun P, Wongsurawat T, Nookaew I, Cheevadhanarak S, et al. *Clostridium manihotivorum* sp. nov., a novel mesophilic anaerobic bacterium that produces cassava pulp-degrading enzymes. PeerJ. 2020 Nov 16;8(e10343):e10343.

58. Jeong CG, Seo BJ, Nazki S, Jung BK, Khatun A, Yang MS, et al. Characterization of *Clostridium novyi* isolated from a sow in a sudden death case in Korea. BMC Vet Res. 2020 May 6;16(1):127.

59. Bettegowda C, Huang X, Lin J, Cheong I, Kohli M, Szabo SA, et al. The genome and transcriptomes of the anti-tumor agent *Clostridium novyi*-NT. Nat Biotechnol. 2006 Dec;24(12):1573–80.

60. Pyne ME, Utturkar S, Brown SD, Moo-Young M, Chung DA, Chou CP. Improved draft genome sequence of *Clostridium pasteurianum* strain ATCC 6013 (DSM 525) using a hybrid next-generation sequencing approach. Genome Announc. 2014 Aug 7;2(4):e00790–14.

61. Poehlein A, Grosse-Honebrink A, Zhang Y, Minton NP, Daniel R. Complete genome sequence of

the nitrogen-fixing and solvent-producing *Clostridium pasteurianum* DSM 525. Genome Announc [Internet]. 2015 Feb 19;3(1). Available from: https://pubmed.ncbi.nlm.nih.gov/25700415/

62. Pyne ME, Liu X, Moo-Young M, Chung DA, Chou CP. Genome-directed analysis of prophage excision, host defence systems, and central fermentative metabolism in *Clostridium pasteurianum*. Sci Rep. 2016 Sep 19;6(1):26228.

63. Sandoval NR, Venkataramanan KP, Groth TS, Papoutsakis ET. Whole-genome sequence of an evolved *Clostridium pasteurianum* strain reveals Spo0A deficiency responsible for increased butanol production and superior growth. Biotechnol Biofuels. 2015 Dec 24;8(1):227.

64. Tigunova O, Samborskyy M, Bratishko V, Balabak O, Zelena L, Shulga S. Main genome characteristics of butanol-producing *Clostridium* sp. UCM B-7570 strain. J Appl Genet. 2023 Sep;64(3):559–67.

65. Sichtig H, Minogue T, Yan Y, Stefan C, Hall A, Tallon L, et al. FDA-ARGOS is a database with public quality-controlled reference genomes for diagnostic use and regulatory science. Nat Commun. 2019 Jul 25;10(1):3313.

66. Myers GSA, Rasko DA, Cheung JK, Ravel J, Seshadri R, DeBoy RT, et al. Skewed genomic variability in strains of the toxigenic bacterial pathogen, *Clostridium perfringens*. Genome Res. 2006 Aug;16(8):1031–40.

67. Lacey JA, Allnutt TR, Vezina B, Van TTH, Stent T, Han X, et al. Whole genome analysis reveals the diversity and evolutionary relationships between necrotic enteritis-causing strains of *Clostridium perfringens*. BMC Genomics. 2018 May 22;19(1):379.

68. Mehdizadeh Gohari I, Kropinski AM, Weese SJ, Parreira VR, Whitehead AE, Boerlin P, et al. Plasmid characterization and chromosome analysis of two netF+ *Clostridium perfringens* isolates associated with foal and canine necrotizing enteritis. PLoS One. 2016 Feb 9;11(2):e0148344.

69. Poehlein A, Hartwich K, Krabben P, Ehrenreich A, Liebl W, Dürre P, et al. Complete genome sequence of the solvent producer *Clostridium saccharobutylicum* NCP262 (DSM 13864). Genome Announc. 2013 Nov 27;1(6):e00997–13.

70. Poehlein A, Krabben P, Dürre P, Daniel R. Complete genome sequence of the solvent producer *Clostridium saccharoperbutylacetonicum* strain DSM 14923. Genome Announc [Internet]. 2014 Oct 16;2(5). Available from: https://journals.asm.org/doi/pdf/10.1128/genomea.01056-14?download=true

71. Zhu Z, Guo T, Zheng H, Song T, Ouyang P, Xie J. Complete genome sequence of a malodorant-producing acetogen, *Clostridium scatologenes* ATCC 25775(T). J Biotechnol. 2015 Oct 20;212:19–20.

72. Thomas P, Abdel-Glil MY, Subbaiyan A, Busch A, Eichhorn I, Wieler LH, et al. First comparative analysis of *Clostridium septicum* genomes provides insights into the taxonomy, species genetic diversity, and virulence related to gas gangrene. Front Microbiol. 2021 Dec 9;12:771945.

73. Wang SX, Park M, Summage-West CV, Reyna M, Kim SG. Genome sequence *of Clostridium septicum* strain WW106, isolated from influent wastewater at a research center with multiple-species research animal facilities. Microbiol Resour Announc. 2024 Jan 17;13(1):e0076823.

74. Kubiak AM, Poehlein A, Budd P, Kuehne SA, Winzer K, Theys J, et al. Complete genome sequence of the nonpathogenic soil-dwelling bacterium *Clostridium sporogenes* strain NCIMB 10696. Genome Announc [Internet]. 2015 Aug 27 [cited 2025 Jun 18];3(4). Available from: http://dx.doi.org/10.1128/genomeA.00942-15

75. Smith TJ, Xie G, Williamson CHD, Hill KK, Fernández RA, Sahl JW, et al. Genomic characterization of newly completed genomes of botulinum neurotoxin-producing species from Argentina, Australia, and Africa. Genome Biol Evol. 2020 Mar 1;12(3):229–42.

76. Chen C, Li L, Huang K, Zhang J, Xie WY, Lu Y, et al. Sulfate-reducing bacteria and methanogens are involved in arsenic methylation and demethylation in paddy soils. ISME J. 2019 Oct;13(10):2523–35.

77. Cambridge JM, Blinkova AL, Salvador Rocha EI, Bode Hernández A, Moreno M, Ginés-Candelaria E, et al. Genomics of *Clostridium taeniosporum*, an organism which forms endospores with ribbon-like appendages. PLoS One. 2018 Jan 2;13(1):e0189673.

78. Bruggemann H, Baumer S, Fricke WF, Wiezer A, Liesegang H, Decker I, et al. The genome sequence of *Clostridium tetani*, the causative agent of tetanus disease. Proc Natl Acad Sci U S A. 2003 Feb 4;100(3):1316–21.

79. Fournier PE, Levy PY, Million M, Croce O, Blanc-Tailleur C, Brouqui P, et al. Genome of a chronic osteitis-causing *Clostridium tetani*. New Microbes New Infect. 2014 Jan 28;2(1):25–6.

80. Shitada C, Sekizuka T, Yamamoto A, Sakamoto C, Hashino M, Kuroda M, et al. Comparative pathogenomic analysis reveals a highly tetanus toxin-producing clade of *Clostridium tetani* isolates in Japan. mSphere. 2023 Dec 20;8(6):e0036923.

81. Koendjbiharie JG, Wiersma K, van Kranenburg R. Investigating the central metabolism of *Clostridium thermosuccinogenes*. Appl Environ Microbiol [Internet]. 2018 Jul 1;84(13). Available from: http://dx.doi.org/10.1128/aem.00363-18

82. Lee J, Jang YS, Han MJ, Kim JY, Lee SY. Deciphering *Clostridium tyrobutyricum* metabolism based on the whole-genome sequence and proteome analyses. MBio. 2016 Jun 14;7(3):e00743–16.

83. Liu T, Zhu L, Zhu Z, Jiang L. Genome Sequence Analysis of *Clostridium tyrobutyricum*, a Promising Microbial Host for Human Health and Industrial Applications. Curr Microbiol. 2020 Nov 1;77(11):3685–94.

84. Wu Q, Liu T, Zhu L, Huang H, Jiang L. Insights from the complete genome sequence of *Clostridium tyrobutyricum* provide a platform for biotechnological and industrial applications. J Ind Microbiol Biotechnol. 2017 Aug;44(8):1245–60.

85. Lou YC, Olm MR, Diamond S, Crits-Christoph A, Firek BA, Baker R, et al. Infant gut strain

persistence is associated with maternal origin, phylogeny, and traits including surface adhesion and iron acquisition. Cell Rep Med. 2021 Sep 21;2(9):100393.
