# Supplementary figures and images for: Flagellar glycosylation with pseudaminic acids is widespread in the genus Clostridium
Source: BMC Microbiol. 2026 May 6;26:574. doi: 10.1186/s12866-026-04975-z (PMC13317340; doi:10.1186/s12866-026-04975-z)

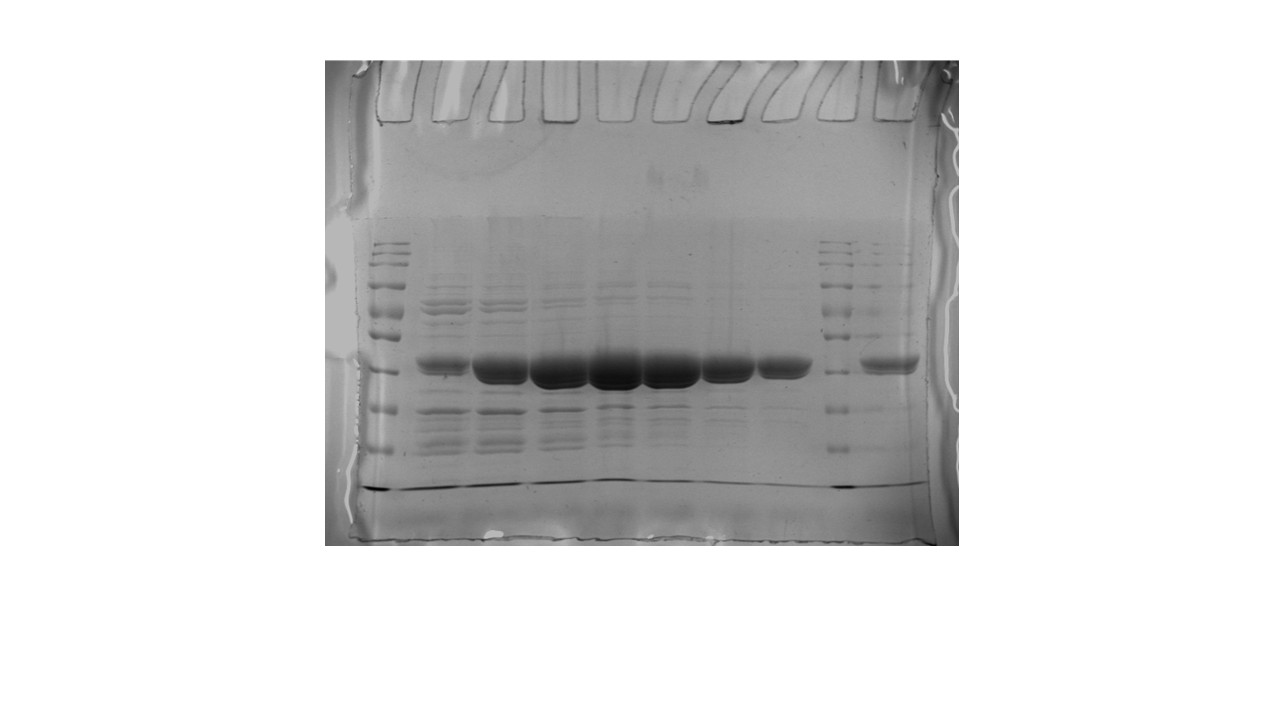

Supplement: Supplementary file 2 — Additional file 2. Original SDS-PAGE gel image for SEC purified PseB [file 12866_2026_4975_MOESM2_ESM.jpg]
